# Optimal types and doses of exercise for improving sleep quality in perinatal women: a systematic review and network meta-analysis based on randomized controlled trials

**DOI:** 10.1186/s12884-026-08673-6

**Published:** 2026-01-22

**Authors:** Longkang Guo, Hengyi Song, Lintao Suo, Jingyuan Yang, Xuehaiyue Lv, Wei Han

**Affiliations:** 1 School of Graduate Education, Shandong Sport University, Jinan, China; 2 School of Competitive Sports, Shandong Sport University, Rizhao, China

**Keywords:** Exercise, Perinatal period, Sleep quality, Meta-analysis, Systematic review

## Abstract

**Background:**

The perinatal period spans from the onset of pregnancy to one year postpartum. Sleep quality is particularly critical during this period. An appropriate exercise regimen can significantly improve their sleep quality. Previous studies have demonstrated that exercise effectively enhances sleep quality; however, the optimal regimen remains unclear. To address this limitation, this study employs a network meta-analysis to systematically evaluate the effects of different types of exercise and dosage (including duration, frequency, and session length) on the sleep quality of perinatal women. This analysis provides evidence-based recommendations to improve sleep quality in perinatal women.

**Methods:**

Using a combination of manual and computer-assisted search strategies, we searched PubMed, Web of Science, EBSCO, Cochrane Library, Scopus, Embase, ProQuest, CNKI, and Wanfang databases to identify randomized controlled trials on the effects of different exercise interventions on the sleep quality of perinatal women. The search was conducted up to August 5, 2025.The risk of bias was assessed using RevMan 5.4.1 software, and traditional meta-analysis and network meta-analysis were conducted using Stata 16.0 software to generate forest plots, network evidence plots, funnel plots, Surface Under the Cumulative Ranking Curve (SUCRA) plots, and GRADE evaluation plots. Results were reported using standardized mean difference (SMD) and 95% confidence intervals (CI).

**Results:**

This study included 23 RCTs with 1,862 participants. Five different types of exercise were compared: Aerobic Exercise (AE), Aerobic Combined With Resistance Training (AE + RT), Yoga, Pilates, and Relaxation Exercises(RE). The highest-ranked intervention was RE [SMD = -2.71, 95% CI (-3.96, -1.45), SUCRA = 88.5]. Four intervention durations (≤ 4 weeks, 5–8 weeks, 9–12 weeks, 13–16 weeks), three frequencies (1–2 times per week, 3 times per week, 5 times per week), and three session durations (≤ 30 min, 30–60 min, > 60 min) were analyzed. Network meta-analysis revealed that RE was relatively effective [SMD = -2.71, 95% CI (-3.96, -1.45), SUCRA = 88.5]. For intervention duration, ≤ 4 weeks showed greater effectiveness [SMD = -3.13, 95% CI (-4.21, -2.05), SUCRA = 97.2]. Regarding frequency, 1–2 times per week was more effective [SMD = -2.54, 95% CI (-3.57, -2.05), SUCRA = 97.7]. For session duration, 30–60 min was the relatively effective [SMD = -2.54, 95% CI (-3.41, -1.67), SUCRA = 99.0].

**Conclusion:**

This network meta-analysis systematically evaluated different exercise types, durations, frequencies, and session lengths on the sleep quality of perinatal women, with a particular focus on the combination of exercise type and dosage. The findings revealed that RE lasting ≤ 4 weeks, performed 1–2 times per week, and 30–60 min per session, was the relatively effective. It is recommended that this regimen be prioritized in perinatal healthcare to optimize sleep and overall health. Future research should further explore the types, dosages, and combinations of exercise to provide more evidence for targeted interventions.

**Supplementary Information:**

The online version contains supplementary material available at 10.1186/s12884-026-08673-6.

## Introduction

Sleep accounts for approximately one-third of the human lifespan and is an essential physiological activity crucial for overall health [1]. Sufficient sleep duration is widely associated with positive effects on cardiovascular health, cognitive function, mental health, physical health, and chronic diseases such as obesity [2]. The perinatal period constitutes a unique physiological phase for women, during which a series of physiological, endocrine, and psychosocial changes occur. Sleep quality is crucial for overall health management during this period. Previous studies have shown that perinatal women are primarily plagued by poor sleep quality and mental health issues [3]. Good sleep can help perinatal women maintain physical and mental well-being, reduce the occurrence of pregnancy-related complications, and support fetal development. On the other hand, sleep disorders in perinatal women can increase the risk of developing conditions such as gestational hypertension, gestational diabetes, and perinatal depression [4]. High-quality sleep can reduce the occurrence of adverse maternal and neonatal outcomes and improve the quality of life for both the mother and the infant [5]. Therefore, sleep quality is crucial for perinatal women. The World Health Organization (WHO) has long defined the perinatal period as a continuous phase of health management covering pregnancy through the postpartum period. Combining the pregnancy and postpartum populations allows for the integration of fragmented research evidence and enhances the statistical power of the analysis [6].

Currently, the methods for improving sleep quality are mainly divided into pharmacological and non-pharmacological treatments. Although pharmacological treatments are effective in improving sleep quality, they may have adverse effects on newborns, as demonstrated in multiple studies.Wikner [7] found in their study that the use of benzodiazepines during pregnancy to improve sleep quality may increase the risk of preterm birth and low birth weight. Korean scholars have found that exposure to benzodiazepines during the first trimester of perinatal is significantly associated with an increased risk of fetal malformations and cardiac defects [8]. Therefore, choosing a non-pharmacological intervention is safer and more reliable for both the mother and the fetus. Physical exercise, as a non-pharmacological intervention, has been shown to effectively improve sleep quality [9–11]. Sonmezer [12] found that Pilates training twice a week for eight weeks significantly improved the sleep quality of perinatal women. Azward et al. found that yoga practiced twice a week for four weeks also improved sleep quality. However, existing studies mostly provide limited data and have not thoroughly explored the specific effects of different exercise types and dosages on the sleep quality of perinatal women.For example, Yang et al. [10] and Choong et al. [13] included seven studies in their respective research, which demonstrated that exercise is effective in improving sleep quality in perinatal women, but no subgroup analysis was conducted. In their meta-analysis of 14 studies, Liu et al. [14] were limited by methodological constraints and could only combine studies with direct comparisons, failing to estimate the effects of interventions that were not directly compared via a common comparator. Perinatal women experience changes in core muscle function and increased cardiovascular load, which demand higher safety and specificity in exercise. It is necessary to conduct multidimensional comparisons to identify the most suitable exercise types and dosages.Therefore, determining the optimal exercise types and dosages is crucial for improving the sleep quality of perinatal women. Traditional pairwise meta-analysis has limitations, including the inability to compare multiple exercise types, a lack of dosage evaluation, and the inability to conduct indirect comparisons.

This study aims to systematically compare the effects of different exercise types (AE, AE + RT, Yoga, Pilates, RE) and exercise doses (intervention duration, weekly frequency, single session duration) on sleep quality in perinatal women using a network meta-analysis. It will rank multiple exercise regimens to provide evidence-based guidance for formulating optimized, individualized exercise prescriptions.

## Materials and methods

This study strictly adheres to the PRISMA guidelines for reporting systematic reviews and meta-analyses [15] and has been registered on the international platform PROSPERO with registration number CRD 420,251,123,472[PROSPERO].

### Literature search strategy

We searched nine databases using both manual and computer-assisted methods: PubMed, Web of Science, EBSCO, the Cochrane Library, Scopus, Embase, ProQuest, CNKI, and Wanfang Database.The search covered the period from the inception of each database until August 5, 2025. A combination of subject terms and free-text terms was used to collect RCTs on the effects of different exercise types on the sleep quality of perinatal women. Detailed search terms are provided in Supplementary table. As an example, the specific search strategy for PubMed is shown in Table 1.

**Table 1:** PubMed search strategy

Steps	Search strategies
#1	(“Exercise“[Mesh]) OR (Exercises) OR (Physical Activity)) OR (Physical Activities)) OR (Motor Activity)) OR (Sport)) OR (Aerobics)) OR (Training)) OR (Trainings)) OR (Jogging)) OR (Walking)) OR (Ambulation)) OR (Yoga)) OR (Swimming)) OR (Dancing)) OR (Cycling)) OR (Resistance)) OR (Pilates)) OR (Stretching)) OR (Tai Chi)) OR (Qigong)) OR (Mindfulness meditation)) OR (gym)) OR (fitness)) OR (workout)) OR (recreation)) OR (cardio)) OR (relaxation)) OR (run)) OR (jog)) OR (strength training)) OR (Resistance training))
#2	((((((“Perinatal Care“[Mesh]) OR (“Pregnancy“[Mesh])) OR (“Prenatal Care“[Mesh])) OR (“Postpartum Period“[Mesh])) OR (“Postnatal Care“[Mesh])) OR (“Peripartum Period“[Mesh])) OR (perinatal) OR (antenatal)) OR (postnatal)) OR (post-natal)) OR (postpartum)) OR (post-partum)) OR (pregnant)) OR (prenatal)) OR (childbearing)) OR (peripartum)) OR (puerperium)) OR (antepartum)) OR (parturition)) OR (childbirth[Title/Abstract])) OR (gestation)) OR (pre-natal))
#3	((((“Sleep“[Mesh]) OR (“Sleep Initiation and Maintenance Disorders“[Mesh])) OR (“Sleep Quality“[Mesh])) OR (“Dyssomnias“[Mesh])) OR ((sleeping) OR (poor sleep)) OR (sleep problem)) OR (sleep disturbance)) OR (dysomnia)) OR (sleep initiation)) OR (sleep maintenance)) OR (sleep disorder)) OR (sleep restriction)) OR (sleep hygiene)) OR (sleep latency)) OR (sleep duration)) OR (sleep efficiency)) OR (daytime dysfunction)) OR (sleep outcome)) OR (sleep characteristics)) OR (sleep quantity)) OR (difficulty falling asleep)) OR (restless sleep)) OR (awakenings)) OR (snoring)) OR (diurnal sleep)) OR (diurnal tiredness)) OR (sleepiness)) OR (sleep dysfunction)) OR (sleep health)) OR (sleep time)) OR (sleep pattern)) OR (sleep parameters))
#4	((randomized controlled trial[Title/Abstract]) OR (randomized[Title/Abstract])) OR (placebo[Title/Abstract])
#5	#1 AND #2 AND #3 AND #4

### Inclusion and exclusion criteria

The inclusion and exclusion criteria for this study were strictly formulated according to the PICOS principle [16].

#### Inclusion criteria

1. Population (P): The perinatal period generally refers to the time from the onset of pregnancy to one year postpartum [17]. Accordingly, the inclusion criteria for participants in this study are as follows: women aged 18 years or older, who are either pregnant (with no restriction on specific gestational age) or within one year after childbirth.
2. Intervention (I): Various types of exercise suitable for perinatal women (Table 2), including AE, AE + RT, Yoga, Pilates, and RE. This classification is based on Prior studies [19].
3. Comparison Group (C): The comparison group is clearly defined as non-exercise interventions, including routine prenatal/postpartum care (e.g., regular check-ups, health education without exercise guidance) or no specific intervention.
4. Outcomes (O): The primary outcome is sleep quality, measured by at least one of the following validated scales: Pittsburgh Sleep Quality Index (PSQI), Self-Reported Stress Scale (SRSS), General Sleep Disturbance Scale (GSDS), or Insomnia Severity Index (ISI). Studies with sleep quality as a secondary outcome are excluded to prioritize high-relevance evidence. All outcome scales are scored in the same direction, where lower scores indicate better sleep quality. We pooled data from four different sleep scales for statistical analysis. Despite differences in their measurement properties and scoring methods, with the PSQI focusing on a comprehensive evaluation of overall sleep quality, the ISI targeting core insomnia symptoms, and the SRSS and GSDS being more closely aligned with clinical manifestations of sleep disorders, we standardized the effect size using the SMD, which eliminated the differences in scoring units and intervals.
5. Study Design (S): Only randomized controlled trials (RCTs) were included, as this design enhances the reliability of the results [23].

**Table 2:** Definitions of exercise types

type	Definition of Exercise Types
AE	Aerobic exercise, also known as endurance exercise, refers to physical activities primarily fueled by aerobic metabolism when there is an adequate supply of oxygen. These activities primarily include walking and others [18].
AE+RT	A comprehensive exercise mode combining aerobic and resistance exercise [19].
Yoga	Yoga is a mind-body practice that combines physical postures, relaxation, and breathing techniques [20].
Pilates	Pilates is a mind-body exercise that focuses on strength, core stability, flexibility, muscle control, posture, and breathing. The practice can be mat-based or involve the use of specialized equipment [21].
RE	The goal of the practice is to increase flexibility, based on static stretching and relaxation exercises, which mainly include progressive muscle relaxation, Benson relaxation, guided imagery, and structured breathing programs [22].

#### Exclusion criteria

1. Non-randomized controlled trial study designs were excluded, including case reports, clinical reports, and conference abstracts.
2. Review articles are excluded.
3. studies with unclear outcome measures or incomplete data were excluded, such as those that do not report any of the three outcome measures or only provide data from either the pre-test or post-test.

#### Classification of exercise dosage

In this study, we classified the dosage of exercise interventions into three dimensions: duration, frequency, and session length, based on previous research findings [24].The specific classification is as follows:

Exercise Duration Classification: The exercise duration was divided into four groups (≤ 4 weeks, 5–8 weeks, 9–12 weeks, 13–16 weeks). This classification is based on prior studies [25]. Furthermore, it ensures the balance of intervals to avoid affecting the accuracy of the network meta-analysis results.

Exercise Frequency Classification: Exercise frequency was classified into three types (1–2 times per week, 3 times per week, 5 times per week). This classification is based on previous studies [25]. Additionally, it follows the ACOG (2015) perinatal exercise guidelines [26], balancing safety and adherence: 1–2 times per week is suitable for individuals with lower physical fitness, 3 times per week is the core frequency recommended by the guidelines, and 5 times per week represents high-frequency intervention. Finally, to ensure the balance of intervals and avoid affecting the accuracy of the network meta-analysis results, this classification was applied.

Exercise Duration Classification: Exercise duration was classified into three intervals (≤ 30 min, 30–60 min, > 60 min). First, the 60-minute duration is recommended as the safety upper limit by the ACOG guidelines [26], distinguishing between safe and Above-threshold durations. Second, this classification is based on previous studies [25]. Finally, we categorized the exercise duration for perinatal women into short, medium, and long durations: ≤30 min as short-duration exercise, 30–60 min as medium-duration exercise, and > 60 min as long-duration exercise.

### Literature screening and data extraction

This study was Performed independently by two researchers. They screened and processed the literature according to the inclusion and exclusion criteria, followed by a cross-checking process. In case of discrepancies between the two, a third researcher facilitated discussions to reach a consensus and resolve the differences prior to data extraction. The extracted data covered various key elements, including publication date, average age, sample size, intervention type, intervention duration, intervention frequency, and session length, among others.

### Risk of bias and GRADE assessment

Two researchers independently assessed the risk of bias (ROB2) for the included studies using the Cochrane risk of bias assessment tool. This tool covers five core domains: (a) Randomization process, (b) Deviations from intended interventions, (c) Missing outcome data, (d) Measurement of the outcome, (e) Selection of the reported result, and one overall bias rating (Overall Bias), which provides a summary rating of the overall risk of bias for the study.The criteria are as follows: the risk of bias for each domain is classified into three levels: low risk of bias, some concerns, and high risk of bias. If the risk of bias assessment for all domains is rated as “low risk,” the overall risk of bias will be considered “low.” If some domains are rated as “some concerns” and no domain is rated as “high risk,” the overall risk of bias will be rated as “some concerns.” If any domain is rated as “high risk,” the overall risk of bias will be classified as “high risk.”

For studies rated as “high risk” of bias, we assessed their impact on the overall analysis results via sensitivity analysis. Specifically, the sensitivity analysis will proceed as follows: studies with “high risk” bias will be excluded, and the meta-analysis will be re-conducted to observe changes in the results. If the analysis results show significant changes after excluding these studies, the accuracy of the results will decline. If the results do not change much, it indicates that the influence of studies with high bias risk on the overall conclusion is negligible, while the analysis results have strong reliability.

For the primary outcome of the network meta-analysis, we employed the “Grading of Recommendations, Assessment, Development, and Evaluations (GRADE)” framework to assess the certainty of the evidence.

### Statistical analysis

This study used Review Manager 5.4.1 to assess the quality of the included studies and conducted network meta-analysis using Stata 16.0. Given the use of different scales across studies, SMD and 95% CI were used as the effect size measures [27]. Specifically, an SMD of 0.2 ≤ SMD < 0.5 is considered a small effect size, 0.5 ≤ SMD < 0.8 is considered a moderate effect size, and an SMD ≥ 0.8 is considered a large effect size. In the network meta-analysis, we will first generate a network evidence plot to show the direct and indirect comparisons between various exercise interventions. If a direct comparison exists between two interventions, the transitivity assumption was tested and consistency was assessed.

The transitivity assumption assumes that the different interventions in the network are comparable, meaning that when the effects of all interventions are compared through a common comparator or pathway, consistency is maintained. To assess whether the transitivity assumption holds, we first compared the baseline characteristics and intervention features across different interventions to ensure their comparability. Additionally, we examined the common comparators between interventions to verify whether all comparisons between interventions are consistent with the transitivity assumption. If there are significant differences in the characteristics of the intervention groups, this may compromise the transitivity of the intervention effects. In such cases, we will further discuss the potential risk of bias [28].

To verify the consistency between direct and indirect evidence in the network, this study employed a three-level inconsistency testing procedure—“global-local-loop”: First, the global inconsistency statistic of the network was calculated. If *P* > 0.05, it indicates no significant overall inconsistency, and a consistency model is used to combine the effects. If *P* ≤ 0.05, it indicates the presence of overall inconsistency, and further local testing is required. Next, the node-splitting method was used to test the combinations of interventions with direct and indirect comparisons in the network. The differences in the SMD and 95% CI between direct and indirect effects were compared. If *P* ≤ 0.05 for any comparison, it suggests significant local inconsistency, and an inconsistency model should be applied to re-combine the effects. The source of inconsistency was clearly reported in the results. Subsequently, for the intervention loops formed in the network, loop residual tests were used to assess the consistency of the evidence within the loops. If the 95% CI of the loop residual does not include 0 and *P* < 0.05, an inconsistency model should be used for further analysis [29].

Finally, by comparing the SUCRA in the cumulative probability plot, the ranking of different interventions was determined. The SUCRA value ranges from 0 to 100, with 100 indicating the relatively most effective intervention and 0 representing the least effective or ineffective one. In other words, the higher the value, the better the intervention effect.

## Results

### Literature screening results

**Fig. 1:**
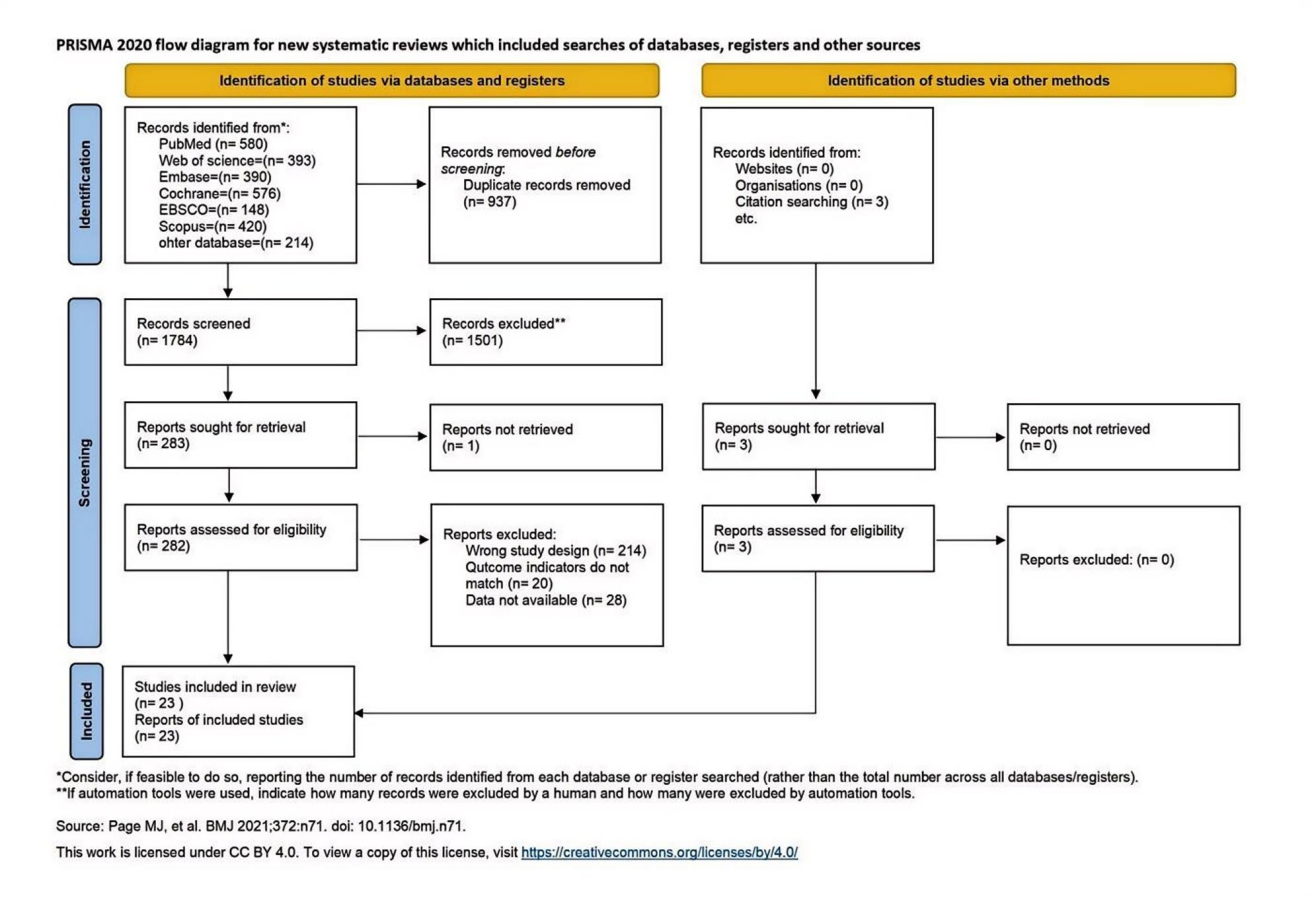
Flowchart of Literature Screening

The literature screening was conducted strictly according to the PRISMA guidelines [30]. A total of 2,724 articles were retrieved from various databases and other sources, including PubMed (580), EBSCO (148), Embase (390), the Cochrane Library (576), Web of Science (393), Scopus (420), ProQuest (145), China National Knowledge Infrastructure (CNKI) (64), and Wanfang Database (5). Furthermore, 3 studies were included from other reviews, resulting in a total of 2,074 identified records. After removing duplicates, 937 records remained. Following a step-by-step screening process, 23 RCTs were ultimately included (Fig. 1).

### Basic characteristics of the included studies

The basic characteristics of the studies included are shown in Table 3 (The table is on pages 43–44). A total of 1,862 participants were enrolled, including 996 in the intervention group and 866 in the control group. The exercise types included AE (*n* = 8), AE + RT (*n* = 3), Yoga (*n* = 2), Pilates (*n* = 7), and RE (*n* = 5). The intervention durations were ≤ 4 weeks, 5–8 weeks, 9–12 weeks, and 13–16 weeks. Intervention frequencies included 1–2 times per week, 3 times per week, and 5 times per week. Intervention session lengths were categorized as ≤ 30 min, 30–60 min, and > 60 min. The primary outcome measures included PSQI, SRSS, GSDS, and ISI. None of the control groups included any exercise type.

**Table 3:** Basic characteristics of the included studies

Study	Country		Sample Size		Age	Duration (weeks)	Exercise category	exercise intervention		Gestational age	Type of the pregnancy	Outcome
Kundarti 2020[31]		Indonesia	C:30 E:30	C:25.1 ± 3.27 E:23.4 ± 3.27		8 weeks	Yoga		Every week/1d/90min	20 weeks- 32 weeks	health	PSQI
Baykan 2025[32]		Turkey	AE:20 RT:20	18–40 years		8 weeks	AE/RE		Every week/2d/20–30 min	12 weeks- 16 weeks	Acute Migraine	PSQI
Yildirim 2023[33]		Turkey	C:17 E:17	C:28.8 ± 5.6 E:30.8 ± 7.0		12 weeks	Pilates		Every week/3d/60min	12 weeks- 24 weeks	lumbar and pelvic pain	PSQI
Mottaghi 2023[34]		Iran	C:27 E:28	C:24.28 ± 5.85 E:24.63 ± 5.55		12 weeks	Pilates		Every week/3d/30min	20 weeks	health	PSQI
Akbas 2015[35]		Turkey	C:26 E:26	C:26.00 ± 4.07 E:28.03 ± 4.45		8 weeks	RE		Every week/3d	27 weeks- 28 weeks	Restless leg syndrome	PSQI
Kim 2022[36]		Korea	C:8 E:8	C:38.14 ± 1.39 E:39.71 ± 2.01		8 weeks	Pilates		Every week/3d/50min	24weeks- 28weeks	health	PSQI
Broberg 2022[37]		Denmark	C:139 E:143	C:31.9 ± 3.7 E:31.9 ± 3.8		12 weeks	RE		Every week/2d/70min	17weeks- 22weeks	Depressed patients	PSQI
Benito 2022[38]		Spain	C:27 E:108	C:31.7 ± 5.36 E:31.92 ± 4.92		12 weeks	AE		Every day/10,000 steps	20 weeks	health	PSQI
Shen 2021[39]		China	C:47 E:51	C:32.8 ± 3.83 E:33.3 ± 4.19		12 weeks	AE		Every week/3d/20min	16weeks- 30weeks	health	PSQI
Mao 2020[40]		China	C:68 E:38	C:27.43 ± 4. 6 E:27.35 ± 4. 37		Until delivery	AE + RT		Unclear	22-29weeks	health	PSQI
Award 2019[41]		Saudi Arabia	C:47 E:51	21–30 years		4 weeks	RE		Every two day/1d/45–60 min	29weeks- 32weeks	health	PSQI
Yang 2018[42]		China	C:65 E:64	C:32.8 ± 3.83 E:33.3 ± 4.19		12 weeks	AE		Every week/3d/15min	Postpartum/6 weeks	health	PSQI
Blanque 2018[43]		Spain	C:67 E:67	C:30.58 ± 4.75 E:32.12 ± 4.43		16 weeks	AE		Every week/3d/60min	20 weeks	health	PSQI
Özkan 2018[44]		Turkey	C:16 E:46	C:27.79 ± 3.90 E:27.93 ± 4.56		4 weeks	RE		Every week/5d/3hours	28weeks- 34weeks	health	PSQI
Shu 2018[45]		China	C:60 E:55	C:29.16 ± 4.46 E:29.29 ± 4.80		4 weeks	Yoga		Every week/1d/20min	10 weeks- 32 weeks	health	SRSS
Shelton 2016[46]		America	C:3 E:3	C:25.0 ± 4.36 E:26.7 ± 2.08		6 weeks	AE		Every week/2d/30min	Postpartum	health	GSDS
Nahid 2015[47]		Iran	C:34 E:33	C:25.64 ± 4.47 E:25.30 ± 4.11		4weeks	RE		Every week/1d/45–60 min	29 weeks- 32 weeks	health	PSQI
Ashrafinia 2014[48]		Iran	C:40 E:40	C:24.4 ± 3.6 E:24.6 ± 3.6		8 weeks	Pilates		Every week/5d/30min	Postpartum	health	PSQI
Mirmohammadali 2012[49]		Iran	C:40 E:40	C:24.4 ± 3.65 E:24.6 ± 3.65		8 weeks	Pilates		Every week/5d	Postpartum	health	PSQI
BA 2010[50]		Nigeria	C:15 E:15	C:31.0 ± 7.1 E:31.8 ± 7.7		6 weeks	AE		Unclear	Unclear	health	ISI
Batool 2024[51]		Pa kistan	AE:19 P:19	C:25.21 ± 2.91 E:24.68 ± 3.23		8 weeks	AE/Pilates		Unclear	26weeks- 32weeks	Health	PSQI
Hyun 2022[52]		Korea	C:7 E:7	C:34.14 ± 3.03 E:34.14 ± 3.82		8 weeks	Pilates		Every week/2d/50min	20weeks- 24weeks	Health	PSQI
Alomarrah 2022[53]		Saudi Arabia	C:45 E:87	31.5 ± 4.3		16 weeks	AE + RT		Every week/3d/60min	≤ 15 weeks	Health	PSQI

Note: Unclear: Not reported in the original studies; Times/week/min: Number of sessions per week and duration per session (minutes)

### Excluded studies summary

A preliminary search through databases and other sources identified 2,724 studies. After deduplication using EndNote X9 software and manual checking, 937 duplicate records were removed, leaving 1,787 studies for initial screening. After screening titles and abstracts, 1,501 records that clearly did not meet the inclusion criteria were excluded, leaving 286 studies for full-text assessment. One study was unavailable in full text, while the remaining 285 studies were carefully assessed. Of these, 214 studies were excluded due to incorrect study design (e.g., Field [54] study combined Tai Chi and yoga as a mixed intervention and pooled the data), 20 studies were excluded due to mismatched outcome indicators (e.g., Chen [55] study used Sleep Characteristics as an indicator, which did not align with the inclusion criteria), and 20 studies were excluded due to unavailable data (e.g., the study by Azward et al. [56] lacked outcome data, only reporting changes in sample size). Ultimately, 23 RCTs were included in the analysis.

### Literature quality assessment and risk of bias evaluation

**Fig. 2:**
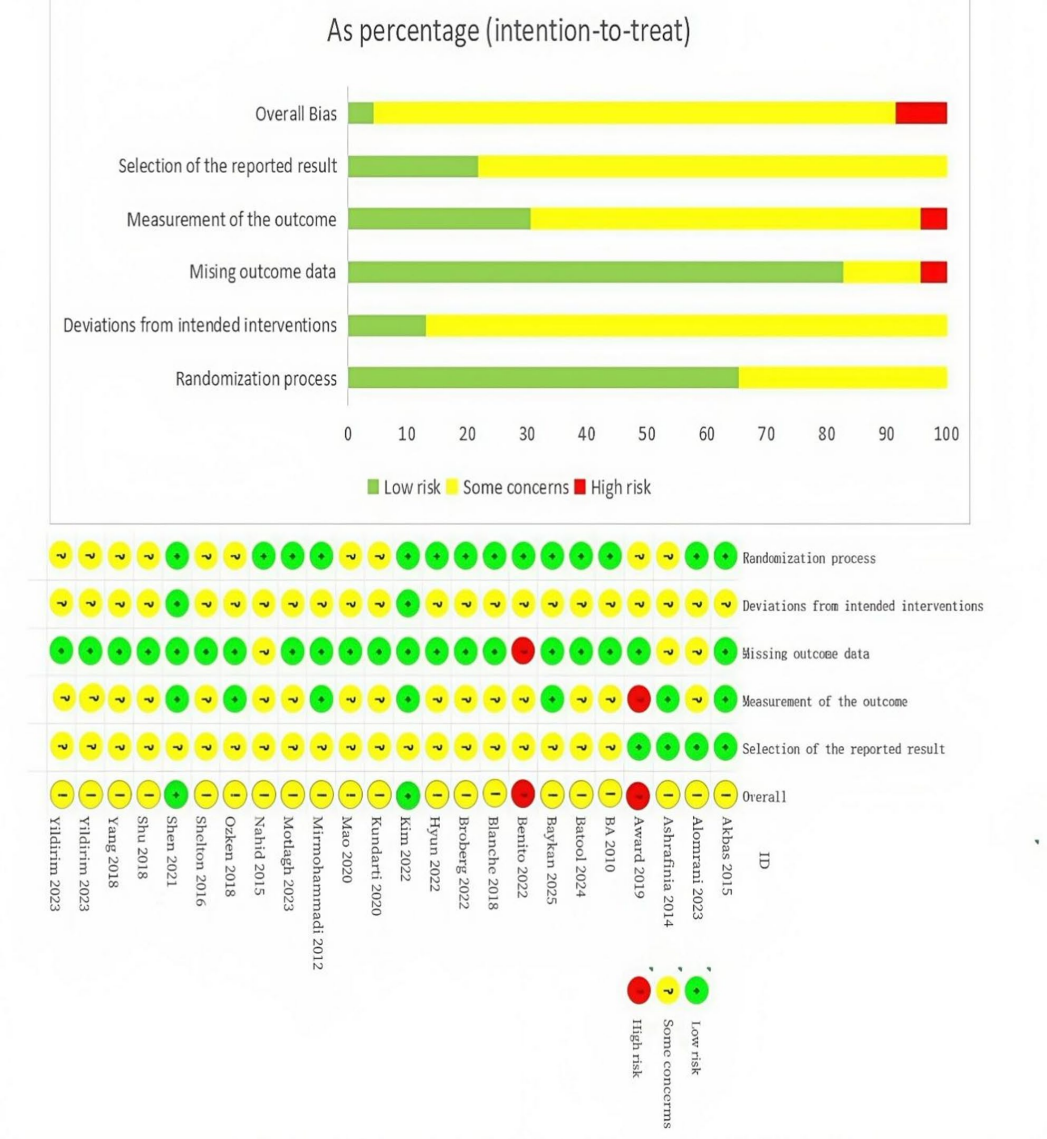
Diagram of Literature Quality Assessment

The quality of the 23 included studies was assessed using the Cochrane ROB 2 risk of bias tool, and the results of the risk of bias evaluation are shown in Fig. 2.

### Traditional meta-analysis

**Fig. 3:**
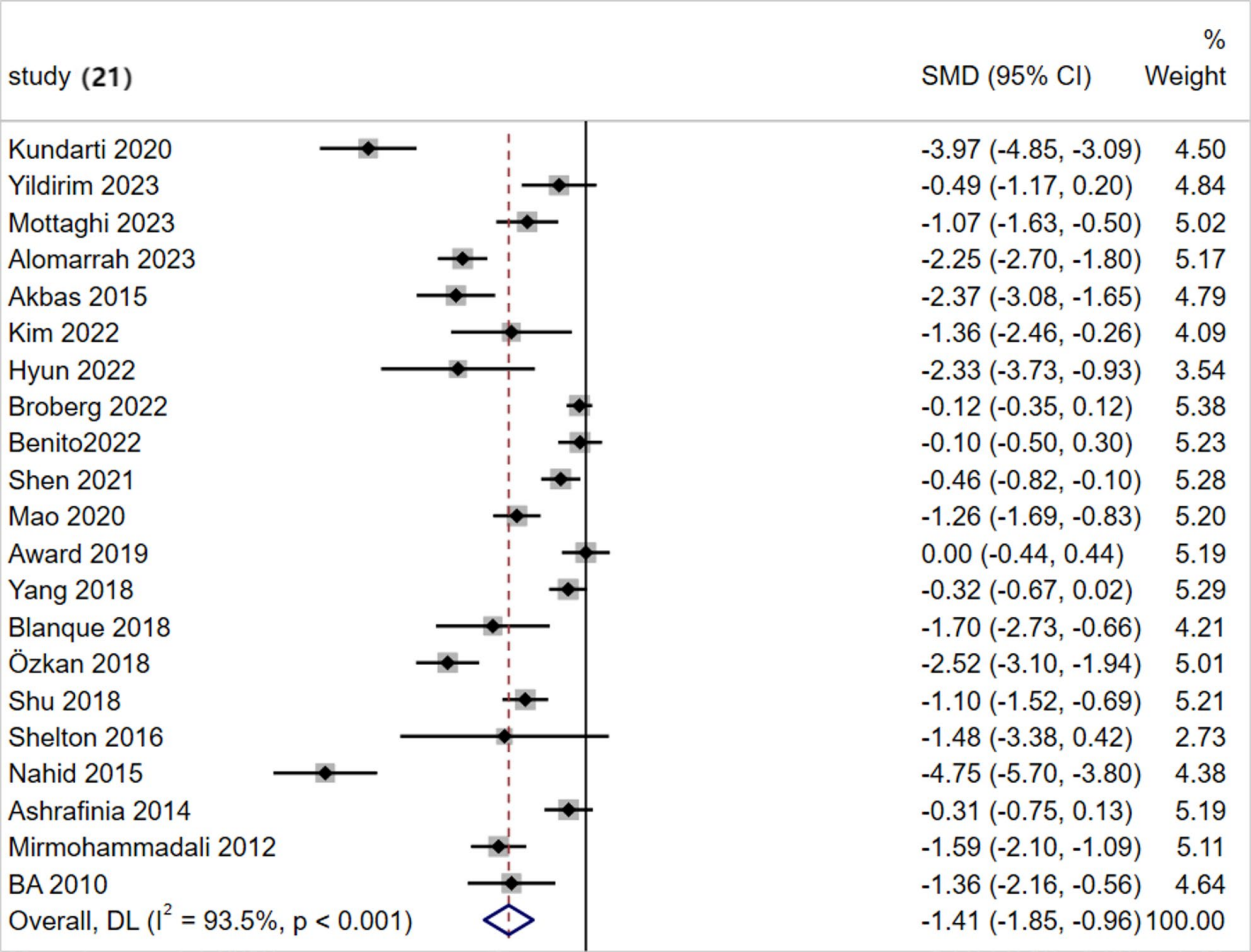
Forest Plot of the Effect of Exercise Interventions on Sleep Quality in Perinatal Women

Given the significant heterogeneity across the 21 included randomized controlled trials (covering five intervention modalities: AE, AE + RT, Yoga, Pilates, and RE), a random-effects model was used to pool the effect sizes. The analysis revealed that the SMD for the effect of exercise intervention on improving sleep quality in pregnant women was − 1.41 (95% CI: −1.85 to −0.96, *p* < 0.001). According to the criteria specified in Sect. 2.5 (where an absolute effect size ≥ 0.8 indicates a large effect), these results demonstrate that, compared with the control group, exercise interventions significantly improve sleep quality in perinatal women(Fig. 3).

#### Sensitivity analysis

**Fig. 4:**
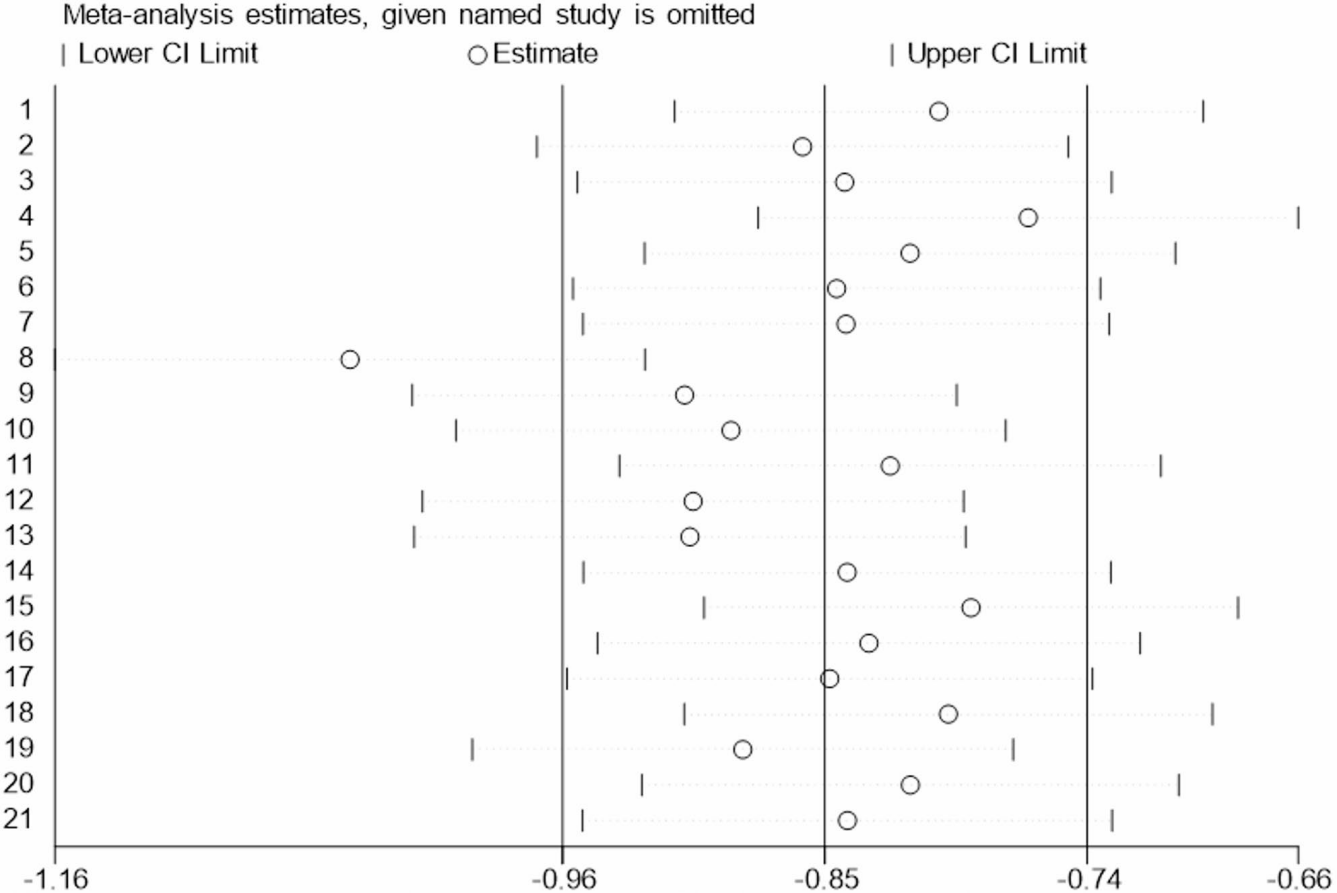
Sensitivity Analysis of the Effect of Exercise on Sleep Quality in Perinatal Women

The results of the sensitivity analysis are presented as a bubble plot (Fig. 4). Using the leave-one-out method, after excluding any single study, the pooled effect size remained negative with a small variation range, and the 95% CI remained entirely within the negative range. This suggests that the analysis results of this study are robust and reliable. Finally, after excluding two high-risk studies, the analysis results remained stable in the negative range, with no significant changes.

#### Meta-Regression analysis

To further explore the sources of heterogeneity, we conducted univariate meta-regression analyses based on the perinatal stage of the study population, health status of the participants, intervention methods, intervention duration, intervention frequency, and session length. The results are shown in Table 4. The meta-regression results indicated that the P-values for the perinatal stage, health status, intervention methods, intervention duration, intervention frequency, session length and sleep measurement tools were all greater than 0.05, suggesting no statistical significance. This indicates that none of these factors significantly contributed to the heterogeneity observed across studies.

**Table 4:** Meta-Regression analysis of heterogeneity factors affecting the effect size on sleep quality in perinatal women

_ES	exp(b)	Std. Err.	t	*P*>|t|	[95% Conf. Interval]
The perinatal stage of the study population					
type	1.987052	1.396617	0.98	0.341	0.4563712,8.651675
_cons	0.1066335	0.0933511	−2.56	0.019	0.0170659,0.666282
The health status of the study population					
type	1.715136	1.33076	0.70	0.495	0.3380816,8.701129
_cons	0.1292249	0.1205198	−2.19	0.041	0.018348,0.9101274
Intervention methods					
type	0.794274	0.1375707	−1.33	0.199	0.5527525,1.141327
_cons	0.4841386	0.2853285	−1.23	0.233	0.1410115,1.662206
Intervention duration, weeks					
type	1.05142	0.2759911	0.19	0.851	0.6057204,1.825074
_cons	0.199252	0.1907009	−1.69	0.109	0.266773,1.48821
Intervention frequency					
type	1.583484	0.6015395	1.21	0.243	0.7104502,3.529343
_cons	0.061479	0.0708435	−2.42	0.027	0.005406,0.699159
Intervention Duration					
type	0.7999432	0.3425621	−0.52	0.609	0.3227013,1.982977
_cons	0.3998652	0.4836401	−0.76	0.460	0.0307868,5.193534
sleep measurement tools					
type	1.043682	0.3974767	0.11	0.912	0.4703141,2.316051
_cons	0.2273543	0.1258837	−2.68	0.015	0.0713519,0.7244372

#### Subgroup analysis

Sleep quality in perinatal women is influenced by multiple factors, including physiological stage, health status, and sleep assessment tools, which may result in inconsistent evaluations of the effects of exercise interventions. To further clarify the applicability of exercise interventions in perinatal women with different characteristics and explore the sources of heterogeneity, this study performed subgroup analyses based on different physiological stages, health statuses, and sleep assessment tools. The aim was to refine applicable scenarios for exercise interventions and provide more precise evidence-based guidance for developing individualized sleep improvement programs.

**Fig. 5:**
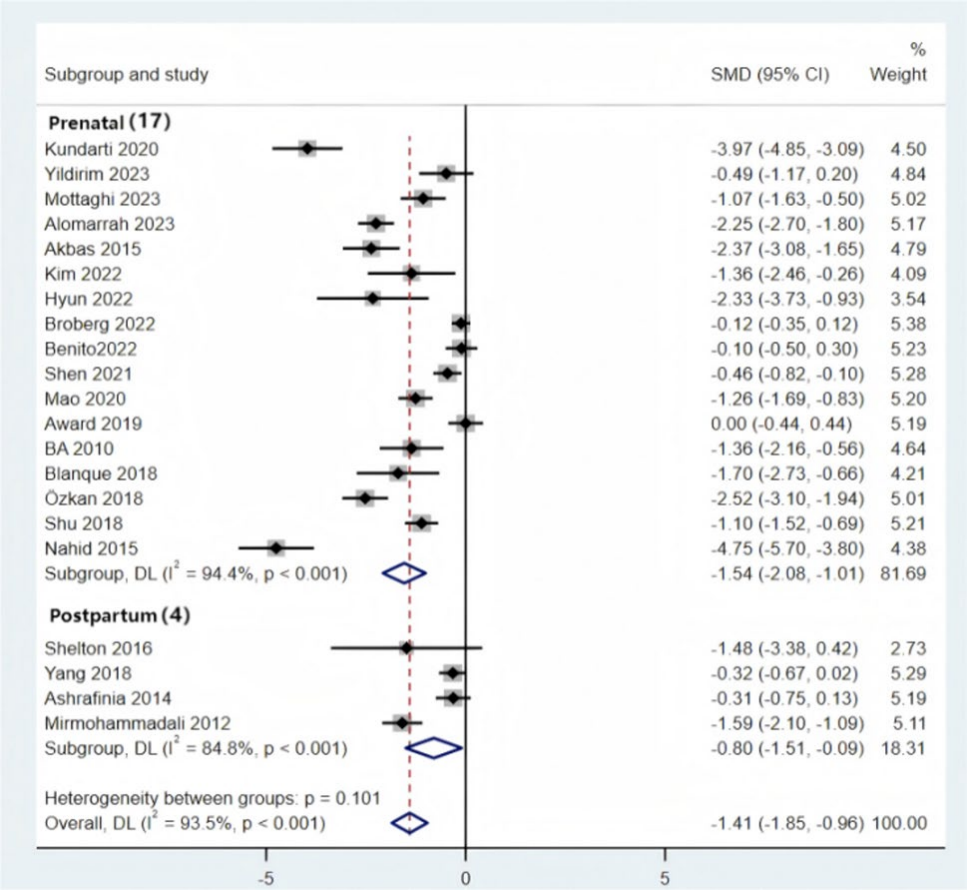
Subgroup Analysis of Pre-Pregnancy and Postpartum Groups

Subgroup analysis was performed by dividing participants into the prepartum and postpartum groups (Fig. 5). The results showed that perinatal exercise interventions significantly improved sleep quality in both subgroups: prepartum [SMD = −1.54, (95% CI = −2.08, −1.01), *P* < 0.001] and postpartum [SMD = −0.80, (95% CI = −1.51, −0.09), *P* < 0.001]. Both effect sizes fall into the “large effect” category (SMD ≥ 0.8), with the prepartum group showing a more pronounced effect.

**Fig. 6:**
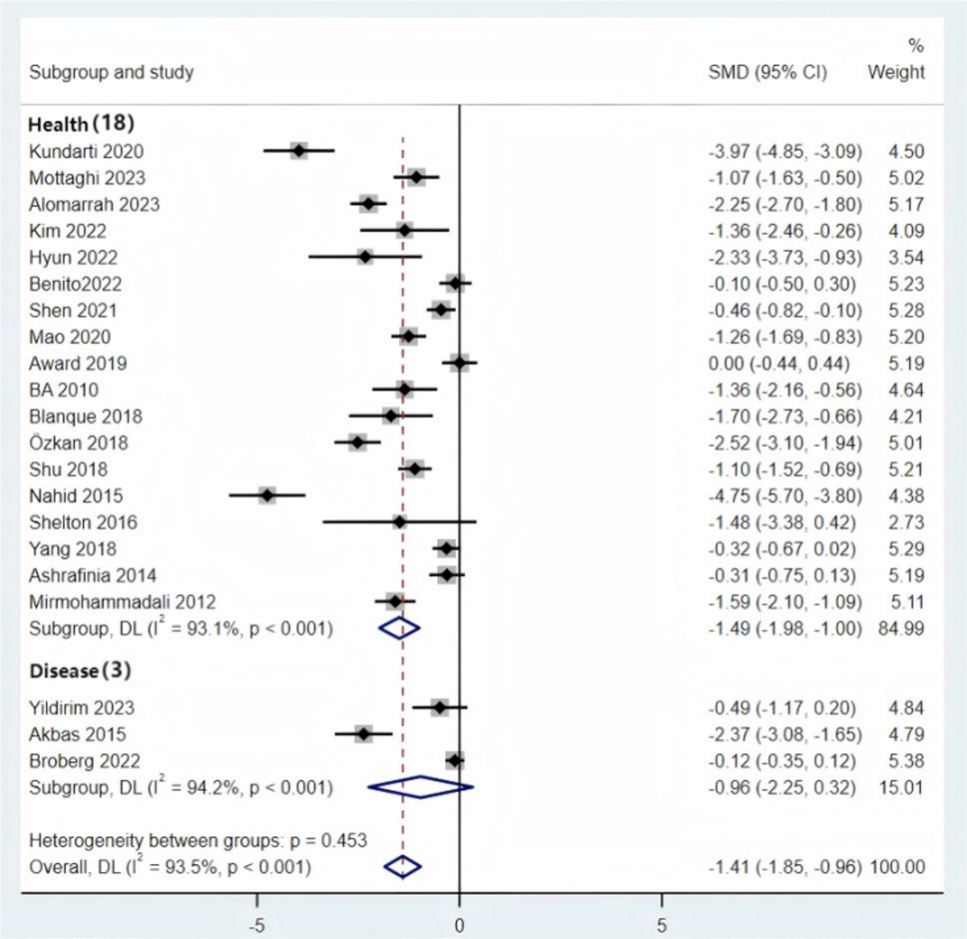
Subgroup Analysis of Healthy Group and Complications Group

Subgroup analysis was performed by dividing participants into healthy and complication groups (Fig. 6). The results showed that perinatal exercise interventions significantly improved the sleep quality of healthy perinatal women [SMD = −1.49, (95% CI = −1.98, −1.00), *P* < 0.001], while the effect was not statistically significant in women with complications [SMD = −0.96, (95% CI = −2.25, 0.32), *P* < 0.001].

**Fig. 7:**
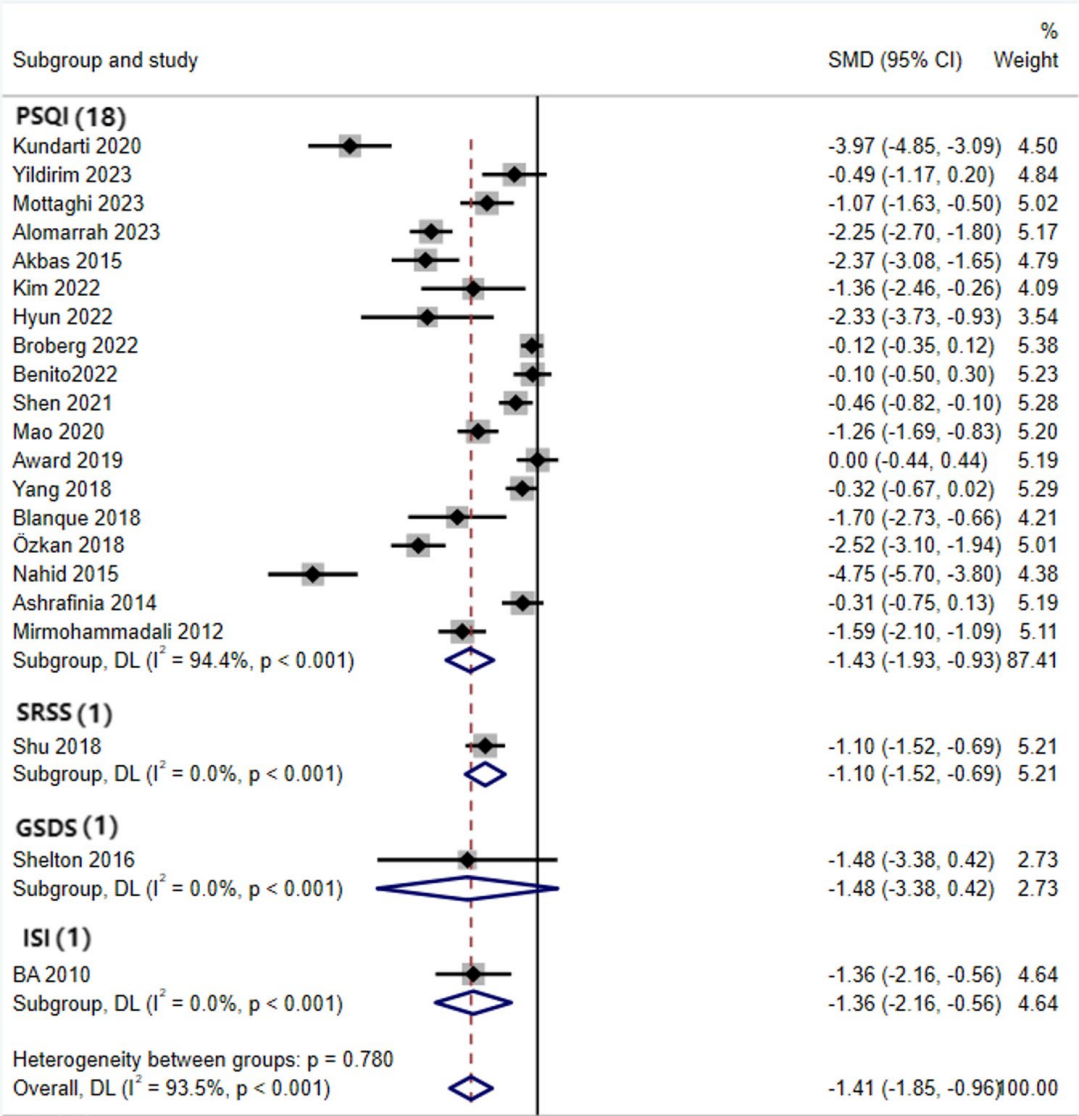
Subgroup Analysis of Sleep Indicators

Subgroup analyses were conducted according to different sleep assessment instruments (Fig. 7). The results indicated that exercise interventions significantly improved sleep outcomes measured by the PSQI [SMD = − 1.43, 95% CI (− 1.93, − 0.93)], SRSS [SMD = − 1.10, 95% CI (− 1.52, − 0.69)] and ISI [SMD = − 1.36, 95% CI (− 2.16, − 0.56)]. However, no statistically significant improvement was observed for sleep outcomes assessed using the GSDS [SMD = − 1.48, 95% CI (− 3.38, 0.42)].

Subgroup analyses revealed that although the P-values for all groups were less than 0.05, no statistically significant differences were observed in the postpartum stage, the group with complications, and the GSDS sleep indicator, which may have had a significant impact on the heterogeneity. Additionally, when comparing different sleep indicators, the SRSS, ISI, and GSDS sleep indicators showed high heterogeneity, likely due to the small number of studies included for these indicators.

### Network Meta-Analysis results

#### Network evidence plot

**Fig. 8:**
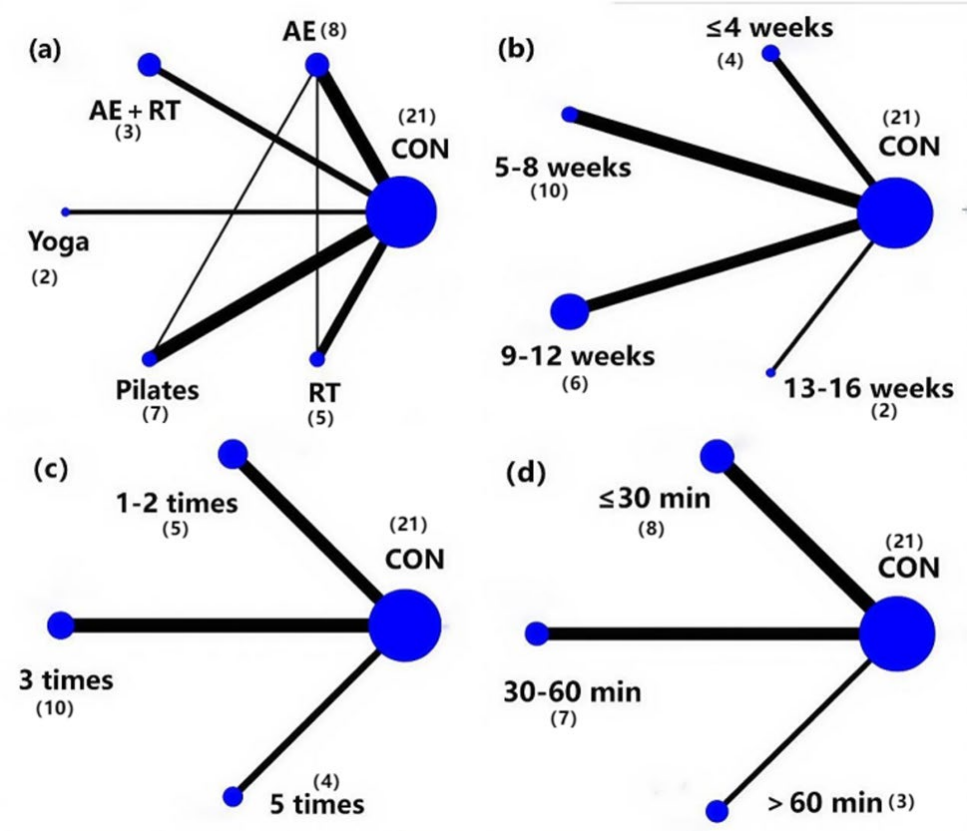
Network Evidence Plot Note: (**a**): Type of exercise; (**b**): Duration in weeks; (**c**): Exercise frequency; (**d**): Exercise duration

The network plot (Fig. 8) shows that different exercise types were studied: AE (*n* = 8), AE + RT (*n* = 3), yoga (*n* = 2), Pilates (*n* = 7), and RE (*n* = 5). Among the 23 included studies, one study did not report the exact duration of the intervention in weeks and was excluded from the analysis comparing different intervention durations [40]. Three studies did not report specific training frequencies and were excluded from the analysis comparing the effects of different frequencies [40, 50, 51]. Five studies did not report the specific duration of training sessions and were excluded from the analysis comparing the effects of different session durations [35, 40, 49–51].Finally, Different intervention durations were as follows: ≤4 weeks in 4 studies, 5–8 weeks in 10 studies, 9–12 weeks in 6 studies, and 13–16 weeks in 2 studies. Intervention frequencies were 1–2 times per week in 5 studies, 3 times per week in 10 studies, and 5 times per week in 4 studies. The duration of each session varied: ≤30 min in 8 studies, 30–60 min in 7 studies, and > 60 min in 3 studies.

#### Transitivity and consistency assumptions

This study provides indirect evidence for the validity of the transitivity assumption in the network meta-analysis by evaluating the consistency of age distribution among participants in the different exercise intervention comparison groups (Supplement figure). Among the 1,862 participants from the 23 studies included, the age distribution was balanced across the different exercise types, and there were no statistically significant differences in the mean age between the groups (*P* > 0.05).

At the same time, consistency assumption testing was performed in this study, and the results are as follows:

The overall inconsistency test for different exercise types showed *P* = 0.6085, *P* > 0.05, indicating good overall consistency.

Further local inconsistency testing was conducted using the node-splitting method, and it was found that all comparisons demonstrated good consistency (*P* > 0.05).

Finally, the consistency within each intervention loop was assessed. It was found that the AE-RE-control loop demonstrated good consistency (*P* > 0.05), while the AE-Pilates-control loop exhibited significant inconsistency (*P* < 0.05), indicating poorer consistency within this loop. Therefore, an inconsistency model was used for the analysis.

The intervention duration, exercise frequency, and session length did not form any loops, so consistency testing is not required.

#### Matrix of network meta-analysis results

**Fig. 9:**
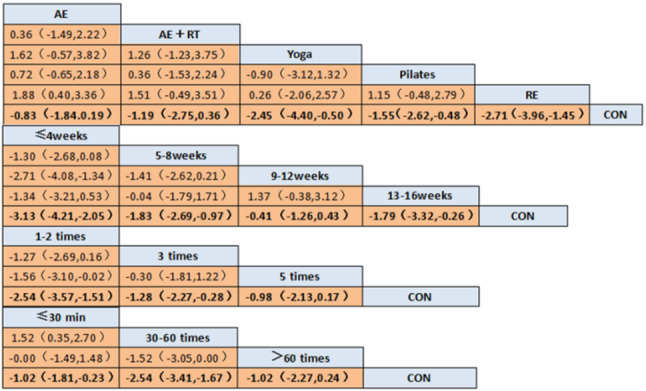
Network Meta Analysis Comparison Matrix

The comparison matrix (Fig. 9) is a core tabular tool that visually presents the “relative effect sizes between all pairs of interventions.“These values represent the pooled average effect sizes derived from both direct and indirect evidence in the network. A negative SMD indicates that the row-defining intervention is superior to the column-defining intervention.”

In terms of exercise types, RE demonstrated the relatively effective intervention [SMD = −2.71, 95% CI (−3.96, −1.45)], followed by yoga [SMD = −2.45, 95% CI (−4.40, −0.50)] and Pilates [SMD = −1.55, 95% CI (−2.62, −0.48)]. However, AE [SMD = −0.83, 95% CI (−1.84, 0.19)]and AE + RT [SMD = −1.19, 95% CI (−2.75, 0.36)] had 95% CI that included “0,” indicating no statistically significant differences.

In terms of intervention duration, training for ≤ 4 weeks demonstrated the relatively effective results [SMD = −3.13, 95% CI (−4.21, −2.05)], followed by 13–16 weeks [SMD = −1.79, 95% CI (−3.32, −0.26)] and 5–8 weeks [SMD = −1.83, 95% CI (−2.69, −0.97)]. However, the intervention effect for 9–12 weeks [SMD = −0.41, 95% CI (−1.26, 0.43)] had a 95% CI that included “0,” indicating no statistically significant difference.

In terms of exercise frequency, training 1–2 times per week demonstrated the relatively effective results [SMD = −2.54, 95% CI (−3.57, −1.51)], followed by training 3 times per week [SMD = −1.28, 95% CI (−2.27, −0.28)]. However, the intervention effect for 5 times per week [SMD = 0.98, 95% CI (−2.13, 0.17)] had a 95% CI that included “0,” indicating no statistically significant difference.

In terms of exercise duration, training for 30–60 min demonstrated the relatively effective results [SMD = −2.54, 95% CI (−3.41, −1.67)], followed by training for < 30 min [SMD = −1.02, 95% CI (−1.81, −0.23)]. However, the intervention effect for > 60 min [SMD = −1.02, 95% CI (−2.27, 0.24)] had a 95% CI that included “0,” indicating no statistically significant difference.

#### Rankings of different exercise types, durations, frequencies, and session lengths

SUCRA refers to the surface under the cumulative ranking curve, where a higher value indicates better intervention effectiveness. The results can be used for indirect ranking of multiple interventions. It is important to note that the SUCRA value does not equate to absolute clinical efficacy; it is simply a statistical measure of the relative ranking of interventions. The SUCRA value represents the relative probability of an intervention’s rank within a given network, rather than its absolute clinical efficacy or effect size.

**Fig. 10:**
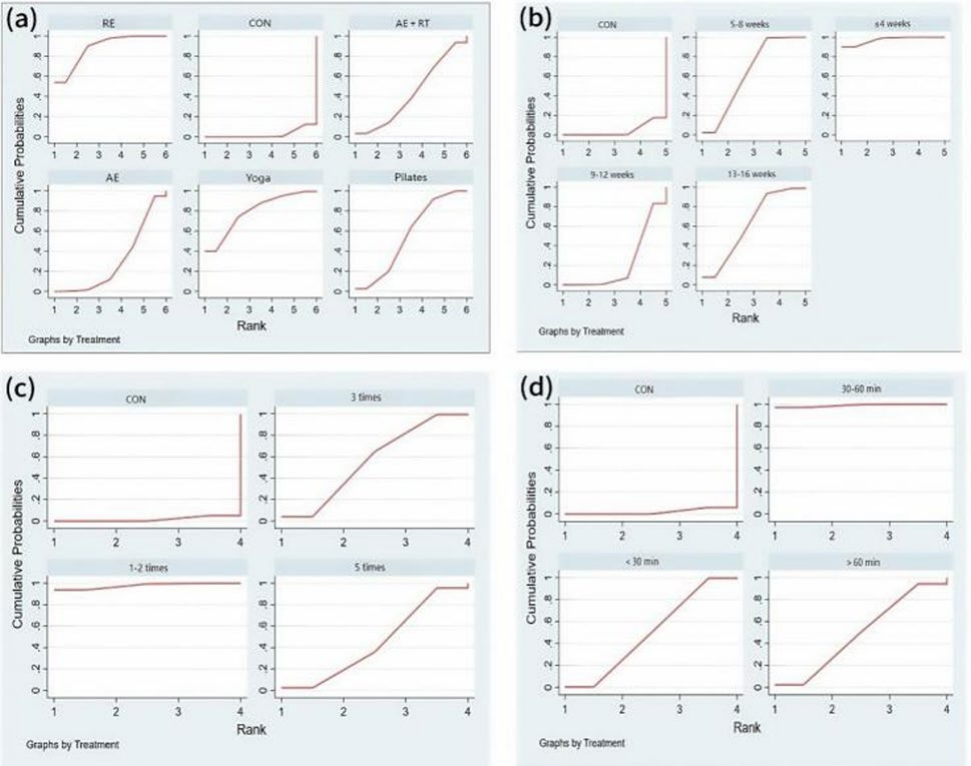
Probability Ranking Plot of the Effects of Different Exercise Interventions Note: (**a**): Type of exercise; (**b**): Duration in weeks; (**c**): Exercise frequency; (**d**): Exercise duration

The SUCRA probability ranking results are shown in Fig. 10. The rankings of the effects of different exercise types on the sleep quality of perinatal women are as follows:

RE(SUCRA = 88.5) > Yoga (SUCRA = 79.2) > Pilates (SUCRA = 55.7) > AE + RT (SUCRA = 43.8) > AE (SUCRA = 30.3).

The ranking of the effects of different exercise frequencies on the sleep quality of perinatal women is as follows: 1–2 times per week (SUCRA = 97.7) > 3 times per week (SUCRA = 56.1) > 5 times per week (SUCRA = 44.5).

The ranking of the effects of different exercise durations on sleep quality is as follows: ≤4 weeks (SUCRA = 97.2) > 5–8 weeks (SUCRA = 63.5) > 13–16 weeks (SUCRA = 62.1) > 9–12 weeks (SUCRA = 22.7).

The ranking of the effects of different intervention durations on the sleep quality of perinatal women is as follows: 30–60 min (SUCRA = 99.0) > ≤ 30 min (SUCRA = 49.9) > Greater than 60 min (SUCRA = 49.1).

#### Publication bias

**Fig. 11:**
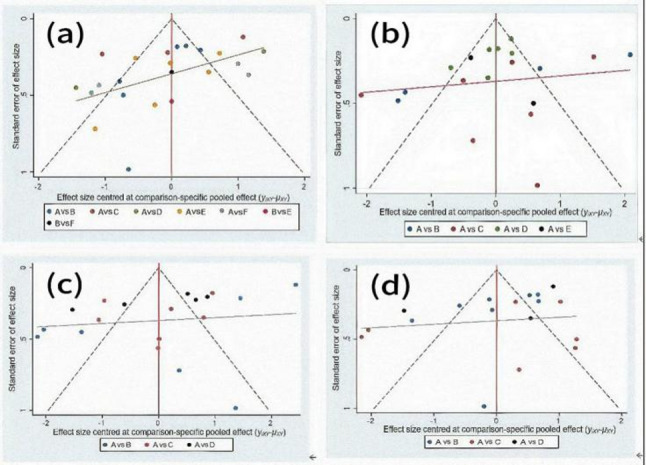
Study Publication Bias Analysis Note: (**a**): Type of exercise; (**b**): Duration in weeks; (**c**): Exercise frequency; (**d**): Exercise duration

The funnel plot is an important tool for assessing publication bias, providing a critical basis for the reliability of the study results [57]. As shown in Fig. 11, the distribution of study points on both sides of the funnel plot is relatively symmetrical, with only 1–2 study points scattered outside the symmetry. This indicates low publication bias among the included studies and that the results are reliable. In addition, publication bias was also tested using the Egger method, yielding a p-value of 0.312 and a 95% CI of (−0.04586783, 1.350525). This indicates that there is no significant publication bias.

#### GRADE evidence quality assessment

**Fig. 12:**
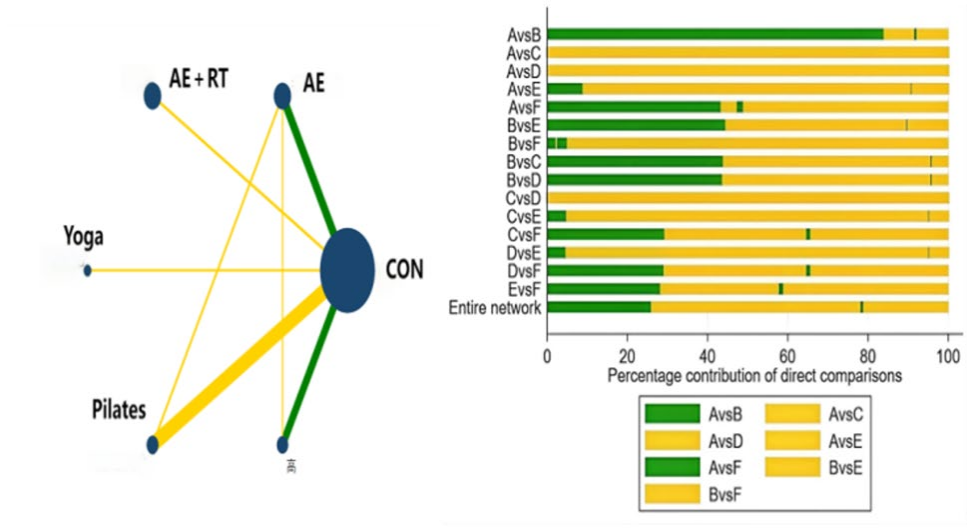
GRADE Evidence Quality Assessment Note: A:Con; B: AE; C: AE + RT; D: Yoga; E: Pilates; F: RE

To ensure the accuracy of the results, this study conducted a GRADE quality assessment for all included studies. As shown in Fig. 12, the majority of comparisons were rated as moderate to low quality in the GRADE assessment. This suggests that, despite some uncertainty and heterogeneity, the conclusions of this study still have a relatively high level of credibility.

## Discussion

Previous studies examining the effects of exercise on sleep quality in perinatal women have relied solely on pairwise meta-analyses. This method can only compare interventions with “direct evidence” and cannot integrate indirect evidence [58]. Given this limitation, this study included 23 randomized controlled trials from 11 countries and a total of 1,862 participants by searching nine databases. It compared the effects of different exercise types and dosages on the sleep quality of perinatal women.According to the SUCRA rankings, RE (SUCRA = 88.5), with a duration of ≤ 4 weeks (SUCRA = 97.2), a frequency of 1–2 times per week (SUCRA = 97.7), and a session length of 30–60 min (SUCRA = 99.0), emerged as a potentially favorable intervention for improving sleep quality in perinatal women.

### Effects of different exercise types on the sleep quality of perinatal women

The results of this study indicate that yoga, Pilates, and RE can all improve the sleep quality of perinatal women. Yoga training indirectly improves sleep quality by reducing anxiety, depression, worry, and fear. Pilates exercises typically lead to increased vagal tone, reduced sympathetic nervous system discharge, and decreased postural heart rate response, along with lower catecholamine levels. These effects help relax the muscles of perinatal women and reduce their reactivity to external stimuli [59]. Compared to other exercise types, Among the included exercise modalities, RE demonstrates a potential advantage in improving sleep quality in perinatal women [SMD = −2.71, 95% CI (−3.96, −1.45), SUCRA = 88.5].

As a relatively effective exercise method for improving the sleep quality of pregnant women during the perinatal period, the beneficial effect of RE on sleep quality in perinatal women may stem from the synergistic effects of physiological regulation and psychological intervention:

In terms of physiological regulation, the core physiological effect of RE lies in the modulation of the autonomic nervous system. Stress and discomfort during the perinatal period can activate the sympathetic nervous system, leading to increased heart rate, rapid breathing, and muscle tension, making it difficult to fall asleep. RE may effectively inhibit the activity of the sympathetic nervous system and activate the antagonistic parasympathetic nervous system. Activation of the parasympathetic nervous system induces a series of physiological changes, including decreased heart rate, reduced blood pressure, and improved digestive function, which guide the body into a deeply relaxed state—a physiological prerequisite for initiating sleep [60]. Studies have shown that RE can improve blood circulation and promote muscle relaxation, thereby alleviating physical discomfort and potentially facilitating restful sleep [60].

From a psychological perspective, RE is an effective tool for managing anxiety and stress during perinatal. They help redirect attention from worries and negative thoughts, reducing overall psychological arousal and breaking the “anxiety-insomnia” vicious cycle [61]. Additionally, sustained relaxation training can enhance emotional regulation and stress coping skills in perinatal women [62].

The analysis indicated that RE demonstrated a potential advantage in improving sleep quality in perinatal women, a finding that partly echoes previous work in this field. Liu et al. in their meta-analysis found that RE (Cohen’s d: −2.74) were more effective than Pilates, yoga, water-based exercises, and AE in improving sleep disturbances in perinatal women [14]. Tan et al. also found that RE can serve as an adjunct intervention to routine prenatal care. Liu et al., in their comparison of non-pharmacological interventions on the sleep quality of perinatal women, found that the relative probability ranking indicated RE was more effective [63]. In contrast, other studies have shown that yoga and Pilates have a significant impact on sleep [64]. This discrepancy may be attributed to the relatively small sample sizes in individual studies. However, when these effects are pooled via meta-analysis, the statistical significance observed in the original studies may become non-significant in the combined analysis due to the substantial increase in the total sample size [14].

This study identified significant inconsistency in the closed loop formed by “AE - Pilates - control” through the ring-specific inconsistency test. This inconsistency may be due to two main factors. First, direct evidence comparing AE and Pilates is limited, with only one study available. Second, within that single study, the pre-post intervention differences were markedly different between the two groups (AE: −7.84 vs. Pilates: −12.53). In the included studies, we observed that some exhibited large effect sizes. A similar phenomenon was reported by Liu et al. in their study on the impact of exercise on sleep disorders in pregnant women [14], which may be related to the characteristics of perinatal women. The perinatal period, as a unique physiological stage for women, involves a series of physiological, endocrine, and psychosocial changes, all of which inevitably affect sleep quality [3]. Since sleep quality often reaches its lowest point during this period, any exercise intervention is likely to yield a substantial improvement.

Both yoga and RE improve sleep in perinatal women through distinct mechanisms. However, RE demonstrated relatively more pronounced benefits, which may be associated with its unique physiological and psychological effects. This result aligns with relatively prior studies and supports the superiority of RE. It also offers valuable guidance for public health policy and clinical practice.

### Effects of different exercise durations, frequencies, and session lengths on the sleep quality of perinatal women

In this analysis, exercise duration, frequency, and session length were all found to be associated with differences in the magnitude of sleep quality improvement in perinatal women. This study suggests that for perinatal women, exercise interventions lasting ≤ 4 weeks may be more effective in improving sleep quality. Similarly, a frequency of 1–2 sessions per week and a session duration of 30–60 min appeared to be associated with greater improvements in sleep outcomes.

Regarding exercise duration, interventions lasting ≤ 4 weeks appear to be associated with greater efficacy in improving sleep quality in perinatal women, a finding that is broadly consistent with some prior research.Although previous meta-analyses have not specifically verified exercise durations of ≤ 4 weeks, Liu in their meta-analysis compared interventions with ≤ 8 weeks and > 8 weeks of exercise duration, and found that ≤ 8 weeks was more beneficial for improving sleep disturbances in perinatal women [14]. In contrast, Li’s study found that an exercise duration of 9–10 weeks was more effective in improving sleep quality [25]. Their study included non-perinatal women with insomnia, a population not experiencing the physiological burdens of the perinatal period (such as uterine enlargement and increased cardiovascular load), who may require a longer period to accumulate the effects of exercise. In contrast, this study focused on “perinatal women,” whose sleep disturbances are often associated with “short-term physiological discomfort” (e.g., mid-perinatal back pain and postpartum stress from newborn care), which may explain the differences observed compared to studies that include a broader population. Mechanistically, some studies suggest that 4 weeks of exercise can significantly reduce serum cortisol levels, while exercise durations of 8 weeks or longer have no significant effect on reducing cortisol. This indicates that short-term, high-frequency RE are more effective at rapidly suppressing the HPA axis [65]. Anxiety is also an important factor affecting sleep. Several randomized controlled trials have shown a 15–20% reduction in anxiety scores (STAI) and a 10–12% decrease in depression scores (EPDS) by the 2nd and 4th weeks. Although improvements persist at 8 weeks or longer, the effect size increases more slowly, and reduced adherence leads to a loss of statistical significance [25]. Therefore, exercise interventions of less than 4 weeks may be more beneficial in improving the sleep quality of perinatal women.

Regarding exercise frequency, the current analysis suggests that a regimen of 1–2 sessions per week is associated with better sleep outcomes. Notably, this observation is not fully consistent with parts of the existing evidence base. Previous studies have suggested that exercise 4 times per week is relatively more effective in improving sleep quality [25]. Possible reasons for this discrepancy include: first, this study focused primarily on perinatal women, and the original studies included in the analysis did not include exercise interventions of 4 times per week. Second, the relatively small sample size may have led to biased results. On the other hand, previous research, through a 10-year follow-up study, found that maintaining exercise 2 times per week is necessary to improve sleep quality [66]. Baykan’s study found that AE + RT training and RE performed twice a week both improved sleep problems in perinatal women [31]. Therefore, exercise 1–2 times per week may be more beneficial for improving the sleep quality of perinatal women.

A preliminary finding of this study was a trend indicating that a session duration of 30–60 min may be associated with greater improvement in sleep quality in perinatal women. However, the relationship between session length and efficacy remains unclear and is inconsistent with certain earlier findings. Some studies suggest that exercise interventions of ≥ 60 min are more effective in improving perinatal sleep quality compared to those of ≤ 60 min [14]. However, only three included studies had an exercise duration of ≤ 60 min, and their sample sizes were generally small, which may have influenced the final results. In their study, LU found that perinatal women who exercised more than 30 min per day had better sleep quality compared to those who exercised less. For perinatal women with sleep disturbances, increasing the daily exercise duration (> 30 min) can improve sleep and, consequently, enhance perinatal outcomes [67]. The American College of Obstetricians and Gynecologists (ACOG) Opinion No. 650 states that perinatal women can engage in moderate-intensity exercise for more than 30 min under controlled temperature conditions, as long as the rise in core body temperature does not exceed 1.5 °C, which is considered within the safe range. Furthermore, regardless of a woman’s prior exercise habits, the fetus can tolerate 30 min of vigorous exercise [26]. Therefore, exercise lasting 30–60 min is more effective in improving the sleep quality of perinatal women.

Although this study identifies a relatively optimal exercise intervention for improving sleep quality in perinatal women via network meta-analysis, it has certain limitations. ①Only published literature was included, and unpublished studies were not searched, which may overestimate the intervention effects due to publication bias. ②The sample sizes are unevenly distributed across various exercise types, durations, frequencies, and session lengths, with some having very small sample sizes (*n* < 20). This may result in insufficient statistical power, thus compromising the reliability of the results. ③Different sleep quality assessment tools were used across studies (e.g., PSQI, SRSS, and GSDS), which may vary in their evaluation criteria and sensitivity, potentially reducing the comparability of the results. Therefore, the heterogeneity between these tools may influence the robustness of our study’s conclusions. ④Significant clinical heterogeneity was observed, which may be due to variations in exercise intensity (not consistently reported), participant health status, and cultural backgrounds across studies. ⑤Residual confounding cannot be completely excluded, as RCTs may not fully control for lifestyle factors (e.g., diet, stress management) that could influence sleep quality.

Future research should expand the literature search scope and conduct large-scale, multi-center RCTs with balanced sample sizes across different exercise types and dose groups to enhance statistical power and improve study comprehensiveness. It is recommended to use standardized assessment tools such as the PSQI to uniformly evaluate sleep quality, standardize the reporting of exercise protocols, and clearly specify exercise intensity parameters. A stratified design based on participants’ health status and cultural background may also help reduce heterogeneity. In RCTs, stricter control of lifestyle factors such as diet and stress management should be implemented. Residual confounding could be addressed through stratified or matched designs, and data recording and reporting processes should be refined to improve research transparency. These steps would further validate the effectiveness of the RE intervention protocol—characterized by a duration of ≤ 4 weeks, a frequency of 1–2 sessions per week, and a session length of 30–60 min—and facilitate a deeper exploration of its underlying mechanisms, thereby providing more reliable evidence-based support for sleep interventions in perinatal women.

### Clinical significance

This study found that RE (≤ 4 weeks, 1–2 times per week, 30–60 min per session) were relatively more effective in improving sleep quality in perinatal women, particularly among healthy perinatal women in Asia and Europe, with high clinical applicability. This regimen significantly improves sleep quality by relaxing the body and mind, regulating the nervous system, and reducing psychological stress in perinatal women. Compared with other high-intensity exercises, RE impose less physiological burden on perinatal women, are safer, and are suitable for women at different stages of the perinatal period, especially those with lower physical fitness or specific complications. Furthermore, RE do not require specialized equipment and are easy to perform, resulting in high adaptability and practical applicability. Therefore, it is recommended to prioritize this regimen in perinatal health management to promote the overall health of pregnant women and maternal-infant health.

## Conclusion

This study used network meta-analysis to systematically evaluate the effects of different exercise types, durations, frequencies, and session lengths on sleep quality in perinatal women, with a focus on the combination of exercise type and dose. The results indicated that RE, performed 1–2 times per week for ≤ 4 weeks with each session lasting 30–60 min, was relatively more effective in improving sleep quality in perinatal women. Based on these findings, it is recommended that this regimen be prioritized in perinatal health management to optimize the sleep quality and overall health of perinatal women.

Furthermore, future research should continue to explore different exercise types, doses, and combinations to inform the development of more targeted intervention programs.

## Supplementary Information

Supplementary Material 1.

Supplementary Material 2.

Supplementary Material 3.

## Data Availability

The data in this study is genuine and reliable, and can provide a reference for future research and practice.

## Abbreviations

AE: Aerobic Exercise
AE + RT: Aerobic Exercise + Resistance training
RE: Relaxation Exercise
PSQI: Pittsburgh Sleep Quality Index
SRSS: Self Reported Stress Scale
ISI: Insomnia Severity Index
GSDS: General Sleep Disturbance Scale
