# Supplementary material for: Optimal types and doses of exercise for improving sleep quality in perinatal women: a systematic review and network meta-analysis based on randomized controlled trials
Source: BMC Pregnancy Childbirth. 2026 Jan 22;26:168. doi: 10.1186/s12884-026-08673-6 (PMC12911199; doi:10.1186/s12884-026-08673-6)
Supplement: Supplementary file 2 — Supplementary Material 2. [file 12884_2026_8673_MOESM2_ESM.docx]

**Supplementary table**

**Table1 Research retrieval methods based on Web of Science**

| **steps** | **Search strategies** **[Search deadline (August 5, 2025)]** |
| --- | --- |
| #1 | (((((((((((((((((((((((((((((((((((((((((((((((((TS=(exercise)) OR TS=(Exercise, Acute)) OR TS=(Acute Exercises)) OR TS=( Exercises, Acute)) OR TS=(Acute Exercise)) OR TS=(Physical Activities)) OR TS=(Activities, Physical)) OR TS=( Physical Activity)) OR TS=(Activity, Physical)) OR TS=( Training)) OR TS=( Trainings, Exercise)) OR TS=(Exercise Training)) OR TS=(Training, Exercise)) OR TS=(Exercise Trainings)) OR TS=(Isometric Exercise))) OR TS=(Exercises, Isometric)) OR TS=(Isometric Exercises)) OR TS=(Exercise, Isometric)) OR TS=( Exercises)) OR TS=( Physical Exercises)) OR TS=(Exercise, Physical)) OR TS=( Exercises, Physical)) OR TS=( Physical Exercise)) OR TS=(Exercise, Aerobic)) OR TS=( Exercises, Aerobic)) OR TS=(Aerobic Exercises)) OR TS=( Aerobic Exercise)) OR TS=(Jogging)) OR TS=(Walking)) OR TS=(Ambulation)) OR TS=(Yoga)) OR TS=(Swimming)) OR TS=(Dancing)) OR TS=(Cycling)) OR TS=(Resistance)) OR TS=(Pilates)) OR TS=(Stretching)) OR TS=(Tai Chi)) OR TS=(Qigong)) OR TS=(Mindfulness meditation)) OR TS=(gym)) OR TS=(fitness)) OR TS=(workout)) OR TS=(recreation)) OR TS=(cardio)) OR TS=(relaxation)) OR TS=(jog)) OR TS=(strength training)) OR TS=(Resistance training) |
| #2 | (((((((((((((((((((((((((((((TS=(Perinatal Care)) OR TS=(Pregnancy)) OR TS=(Prenatal Care)) OR TS=(Postpartum Period)) OR TS=(Postnatal Care)) OR TS=(Peripartum Period)) OR TS=(Care, Perinatal)) OR TS=(Gestation)) OR TS=( Pregnancies)) OR TS=( Care, Antenatal)) OR TS=( Antenatal Care)) OR TS=(Puerperium)) OR TS=(Care, Postnatal))) OR TS=(Postpartum Program)) OR TS=(Peripartum Periods)) OR TS=(Peripartums)) OR TS=(Peripartum)) OR TS=(pre-natal)) OR TS=(gestation)) OR TS=(childbirth)) OR TS=(parturition))) OR TS=(childbearing)) OR TS=(prenatal)) OR TS=(pregnant)) OR TS=(post-partum)) OR TS=(post-natal)) OR TS=(postnatal)) OR TS=(antenatal) |
| #3 | ((((((((((((((((((((((((((((((((((((((TS=(Sleep)) OR TS=(Sleep Initiation and Maintenance Disorders)) OR TS=(Sleep Quality)) OR TS=(Dyssomnias)) OR TS=(sleeping)) OR TS=(poor sleep)) OR TS=(sleep problem)) OR TS=(sleep disturbance)) OR TS=(dysomnia)) OR TS=(sleep initiation)) OR TS=(sleep maintenance)) OR TS=(sleep disorder)) OR TS=(sleep restriction)) OR TS=(sleep hygiene)) OR TS=(sleep latency)) OR TS=(sleep duration)) OR TS=(sleep efficiency)) OR TS=(daytime dysfunction)) OR TS=(sleep outcome)) OR TS=(sleep characteristics)) OR TS=(sleep quantity)) OR TS=(difficulty falling asleep)) OR TS=(restless sleep)) OR TS=(awakenings)) OR TS=(snoring)) OR TS=(diurnal sleep)) OR TS=(diurnal tiredness)) OR TS=(sleepiness)) OR TS=(sleep dysfunction)) OR TS=(sleep health)) OR TS=(sleep time)) OR TS=((sleep pattern)) OR TS=(sleep parameters)) OR TS=( Insomnia)) OR TS=(Insomnias)) OR TS=(DIMS)) OR TS=( Sleep Initiation Dysfunctions)) OR TS=(Early Awakening)) OR TS=(Sleeplessness)) |
| #4 | ((TS=(randomized controlled trial)) OR TS=(randomized)) OR TS=(placebo) |
| #5 | A#1 AND #2 AND #3 AND#4 |

**Table 2 Search strategies of Embase**

| **steps** | **Search strategies [Search deadline (August 5, 2025)]** |
| --- | --- |
| #1 | 'exercise, acute':ab,ti OR 'acute exercises':ab,ti OR 'exercises, acute':ab,ti OR 'acute exercise':ab,ti OR 'physical activities':ab,ti OR 'activities, physical':ab,ti OR 'physical activity':ab,ti OR 'activity, physical':ab,ti OR 'trainings, exercise':ab,ti OR 'exercise training':ab,ti OR 'training, exercise':ab,ti OR 'exercise trainings':ab,ti OR 'isometric exercise':ab,ti OR 'exercises, isometric':ab,ti OR 'isometric exercises':ab,ti OR 'exercise, isometric':ab,ti OR 'exercises':ab,ti OR 'physical exercises':ab,ti OR 'exercise, physical':ab,ti OR 'exercises, physical':ab,ti OR 'physical exercise':ab,ti OR 'sprint interval training':ab,ti OR 'exercise, aerobic':ab,ti OR 'exercises, aerobic':ab,ti OR 'aerobic exercises':ab,ti OR 'aerobic exercise':ab,ti OR 'jogging':ab,ti OR 'walking':ab,ti OR 'ambulation':ab,ti OR 'yoga':ab,ti OR 'swimming':ab,ti OR 'dancing':ab,ti OR 'cycling':ab,ti OR 'resistance':ab,ti OR 'pilates':ab,ti OR 'stretching':ab,ti OR 'tai chi':ab,ti OR 'qigong':ab,ti OR 'mindfulness meditation':ab,ti OR 'gym':ab,ti OR 'fitness':ab,ti OR 'workout':ab,ti OR 'recreation':ab,ti OR 'cardio':ab,ti OR 'relaxation':ab,ti OR 'jog':ab,ti OR 'strength training':ab,ti OR 'resistance training':ab,ti OR 'warm up ':ab,ti OR 'calisthenics ':ab,ti OR 'aquatic exercise  ':ab,ti OR 'training':ab,ti |
| #2 | 'care, perinatal':ab,ti OR 'pregnancies':ab,ti OR 'care, antenatal':ab,ti OR 'care, prenatal':ab,ti OR 'antenatal care':ab,ti OR 'women, postpartum':ab,ti OR 'postpartum women':ab,ti OR 'postpartum':ab,ti OR 'period, postpartum':ab,ti OR 'puerperium':ab,ti OR 'care, postnatal':ab,ti OR 'postpartum care':ab,ti OR 'adolescent overweight':ab,ti OR 'postpartum program':ab,ti OR 'peripartum periods':ab,ti OR 'peripartums':ab,ti OR 'peripartum women':ab,ti OR 'peripartum; period':ab,ti OR 'pre-natal':ab,ti OR 'gestation':ab,ti OR 'childbirth':ab,ti OR 'parturition':ab,ti OR 'antepartum':ab,ti OR 'peripartum':ab,ti OR 'childbearing':ab,ti OR 'prenatal':ab,ti OR 'pregnant':ab,ti OR 'post-partum':ab,ti OR 'post-natal':ab,ti OR 'postnatal':ab,ti OR 'antenatal':ab,ti |
| #3 | 'sleeping':ab,ti OR 'sleep habit':ab,ti OR 'habits, sleep':ab,ti OR 'sleeplessness':ab,ti OR 'insomnia disorders':ab,ti OR 'insomnia disorder':ab,ti OR 'insomnias':ab,ti OR ' sleep initiation and maintenance disorders':ab,ti OR 'secondary insomnia':ab,ti OR 'insomnia, secondary':ab,ti OR 'transient insomnia':ab,ti OR 'insomnia, rebound':ab,ti OR 'psychophysiological insomnia':ab,ti OR 'disorders of initiating and maintaining sleep':ab,ti OR 'dims':ab,ti OR 'insomnia, primary':ab,ti OR 'early awakening':ab,ti OR 'primary insomnia':ab,ti OR 'chronic insomnia':ab,ti OR 'sleep initiation dysfunction':ab,ti OR 'dysfunction, sleep initiation':ab,ti OR ' sleep initiation dysfunctions':ab,ti OR 'nonorganic insomnia':ab,ti OR 'quality, sleep':ab,ti OR 'sleep qualities':ab,ti OR 'qualities, sleep':ab,ti OR 'adjustment sleep disorder':ab,ti OR 'sleep disorder':ab,ti OR 'sleep disorder, limit-setting':ab,ti OR 'sleep disorders, extrinsic':ab,ti OR 'environmental sleep disorder':ab,ti OR 'poor sleep':ab,ti OR 'sleep problem':ab,ti OR 'sleep disturbance':ab,ti OR 'sleep initiation':ab,ti OR 'sleep maintenance':ab,ti OR 'sleep restriction':ab,ti OR 'sleep hygiene':ab,ti OR 'sleep latency':ab,ti OR 'sleep duration':ab,ti OR 'sleep efficiency':ab,ti OR 'daytime dysfunction':ab,ti OR 'sleep outcome':ab,ti OR '(sleep characteristics':ab,ti OR 'difficulty falling asleep':ab,ti OR 'restless sleep':ab,ti OR 'awakenings':ab,ti OR 'snoring':ab,ti OR 'diurnal sleep':ab,ti OR 'diurnal tiredness':ab,ti OR 'sleepiness':ab,ti OR 'sleep dysfunction':ab,ti OR 'sleep health':ab,ti OR 'sleep time':ab,ti OR 'sleep pattern':ab,ti OR 'sleep parameters':ab,ti OR 'dyssomnias':ab,ti |
| #4 | 'randomized controlled trial':ab,ti OR ' randomized ':ab,ti OR ' placebo |
| #5 | A#1 AND #2 AND #3 AND#4 |

**Table 3 Search strategies of Cochrane Library**

| **steps** | **Search strategies [Search deadline (August 5, 2025)]** |
| --- | --- |
| #1 | (Exercise, Acute):ti,ab,kw or (Acute Exercises):ti,ab,kw or ( Exercises, Acute):ti,ab,kw or (Acute Exercise):ti,ab,kw or (Physical Activities):ti,ab,kw or (Activities, Physical):ti,ab,kw or ( Physical Activity):ti,ab,kw or (Activity, Physical):ti,ab,kw or ( Trainings, Exercise):ti,ab,kw or (Exercise Training):ti,ab,kw or (Training, Exercise):ti,ab,kw or (Exercise Trainings):ti,ab,kw or (Isometric Exercise):ti,ab,kw or (Exercises, Isometric):ti,ab,kw or (Isometric Exercises):ti,ab,kw or (Exercise, Isometric):ti,ab,kw or ( Exercises):ti,ab,kw or ( Physical Exercises):ti,ab,kw or (Exercise, Physical):ti,ab,kw or ( Exercises, Physical):ti,ab,kw or ( Physical Exercise):ti,ab,kw or (Exercise, Aerobic):ti,ab,kw or ( Exercises, Aerobic):ti,ab,kw or (Aerobic Exercises):ti,ab,kw or ( Aerobic Exercise):ti,ab,kw or (Jogging):ti,ab,kw or (Walking):ti,ab,kw or (Ambulation):ti,ab,kw or (Yoga):ti,ab,kw or (Swimming):ti,ab,kw or (Dancing):ti,ab,kw or (Cycling):ti,ab,kw or (Resistance):ti,ab,kw or (Pilates):ti,ab,kw or (Stretching):ti,ab,kw or (Tai Chi):ti,ab,kw or (Qigong):ti,ab,kw or (Mindfulness meditation):ti,ab,kw or (gym):ti,ab,kw or (workout):ti,ab,kw or (fitness):ti,ab,kw or (recreation):ti,ab,kw or (cardio):ti,ab,kw or (relaxation):ti,ab,kw or (jog):ti,ab,kw or (strength training):ti,ab,kw or (Resistance training):ti,ab,kw |
| #2 | (Care, Perinatal):ti,ab,kw or (Gestation):ti,ab,kw or ( Pregnancies):ti,ab,kw or ( Care, Antenatal):ti,ab,kw or ( Antenatal Care):ti,ab,kw or (Care, Prenatal):ti,ab,kw or (Women, Postpartum):ti,ab,kw or (Postpartum Women):ti,ab,kw or (Postpartum):ti,ab,kw or (Period, Postpartum):ti,ab,kw or (Puerperium):ti,ab,kw or (Postpartum Care):ti,ab,kw or (Care, Postnatal):ti,ab,kw or (Postpartum Program):ti,ab,kw or (Peripartum Periods):ti,ab,kw or (Peripartum; Period):ti,ab,kw or (Peripartums):ti,ab,kw or (Peripartum Women):ti,ab,kw or (pre-natal):ti,ab,kw or (gestation):ti,ab,kw or (childbirth):ti,ab,kw or (antepartum):ti,ab,kw or (parturition):ti,ab,kw or (peripartum):ti,ab,kw or (childbearing):ti,ab,kw or (pregnant):ti,ab,kw or (prenatal):ti,ab,kw or (post-partum):ti,ab,kw or (post-natal):ti,ab,kw or (postnatal):ti,ab,kw or (antenatal):ti,ab,kw |
| #3 | (Sleeping):ti,ab,kw or (Sleep Habit):ti,ab,kw or (Habits, Sleep):ti,ab,kw or (Sleeplessness):ti,ab,kw or (Insomnia Disorders):ti,ab,kw or (Insomnias):ti,ab,kw or (Insomnia Disorder):ti,ab,kw or (Secondary Insomnia):ti,ab,kw or ( Insomnia):ti,ab,kw or (Insomnia, Secondary):ti,ab,kw or (Transient Insomnia):ti,ab,kw or (Insomnia, Rebound):ti,ab,kw or (Psychophysiological Insomnia):ti,ab,kw or ( Disorders of Initiating and Maintaining Sleep):ti,ab,kw or (DIMS):ti,ab,kw or (Early Awakening):ti,ab,kw or ( Insomnia, Primary):ti,ab,kw or (Primary Insomnia):ti,ab,kw or (Chronic Insomnia):ti,ab,kw or (Sleep Initiation Dysfunction):ti,ab,kw or (Dysfunction, Sleep Initiation):ti,ab,kw or ( Sleep Initiation Dysfunctions):ti,ab,kw or (Nonorganic Insomnia):ti,ab,kw or (Quality, Sleep):ti,ab,kw or (Sleep Qualities):ti,ab,kw or (Qualities, Sleep):ti,ab,kw or (Adjustment Sleep Disorder):ti,ab,kw or (Sleep Disorder):ti,ab,kw or (Sleep Disorder, Limit-Setting):ti,ab,kw or ( Sleep Disorders, Extrinsic):ti,ab,kw or ( Sleep Disorders, Extrinsic):ti,ab,kw or (Environmental Sleep Disorder):ti,ab,kw or (poor sleep):ti,ab,kw or (sleep problem):ti,ab,kw or (sleep disturbance):ti,ab,kw or (sleep initiation):ti,ab,kw or (sleep maintenance):ti,ab,kw or (sleep restriction):ti,ab,kw or (sleep hygiene):ti,ab,kw or (sleep latency):ti,ab,kw or (sleep duration):ti,ab,kw or (sleep efficiency):ti,ab,kw or (daytime dysfunction):ti,ab,kw or (sleep characteristics):ti,ab,kw or (sleep outcome):ti,ab,kw or (difficulty falling asleep):ti,ab,kw or (restless sleep):ti,ab,kw or (awakenings):ti,ab,kw or (snoring):ti,ab,kw or (diurnal sleep):ti,ab,kw or (diurnal tiredness):ti,ab,kw or (sleepiness):ti,ab,kw or (sleep health):ti,ab,kw or (sleep dysfunction):ti,ab,kw or (sleep time):ti,ab,kw or (sleep parameters):ti,ab,kw or (sleep pattern) |
| #4 | (randomized controlled trial):ti,ab,kw or (randomized):ti,ab,kw or (placebo):ti,ab,kw |
| #5 | A#1 AND #2 AND #3 AND#4 |

**Table 4 Search strategies of Scopus**

| **steps** | **Search strategies [Search deadline (August 5, 2025)]** |
| --- | --- |
| #1 | Exercise OR Exercises OR "Physical Activity" OR "Physical Activities "OR "Motor Activity" OR Sport OR Aerobics OR Training OR Trainings OR Jogging OR Walking OR Ambulation OR Yoga OR Swimming OR Dancing OR Cycling OR Resistance OR Pilates OR Stretching OR "Tai Chi" OR Qigong OR" Mindfulness meditation" OR gym OR fitness OR workout OR recreation OR cardio OR relaxation OR run OR jog OR "strength training" OR "Resistance training" |
| #2 | ("Perinatal Care" OR"Pregnancy" OR" Prenatal Care"OR "Postpartum Period "OR "Postnatal Care" OR "Peripartum Period "OR perinatal OR antenatal OR postnatal OR post-natal OR postpartum OR post-partum OR pregnan OR pregnant OR prenatal OR childbearing OR peripartum OR puerperium OR antepartum OR parturition OR childbirth OR gestation OR pre-natal) |
| #3 | (Sleep OR "Sleep Initiation and Maintenance Disorders"OR "Sleep Quality"OR Dyssomnias OR sleeping OR dysomnia OR "daytime dysfunction" OR "difficulty falling asleep" OR awakenings OR snoring OR"diurnal tiredness"OR sleepiness OR "sleep dysfunction" OR "sleep health"OR "sleep time" OR "sleep pattern"OR "sleep parameters"OR "poor sleep" OR "sleep problem" OR "sleep disturbance"OR "sleep initiation" OR "sleep maintenance"OR "sleep disorder"OR "sleep restriction" OR "sleep hygiene" OR "sleep latency" OR "sleep duration" OR "sleep efficiency") |
| #4 | ("randomized controlled trial"OR randomized OR placebo) |
| #5 | A#1 AND #2 AND #3 AND#4 |

**Table 5 Search strategies of EBSCO**

| **steps** | **Search strategies [Search deadline (August 5, 2025)]** |
| --- | --- |
| #1 | AB Exercise, Acute OR AB Acute Exercises OR AB Exercises, Acute OR AB Acute Exercise OR AB Physical Activities OR AB Activities, Physical OR AB Physical Activity OR AB Activity, Physical OR AB Trainings, Exercise OR AB Exercise Training OR AB Training, Exercise OR AB Exercise Trainings OR AB Isometric Exercise OR AB Exercises, Isometric OR AB Isometric Exercises OR AB Exercise, Isometric OR AB Exercises OR AB Physical Exercises OR AB Exercise, Physical OR AB Exercises, Physical OR AB Physical Exercise OR AB Exercise, Aerobic OR AB Exercises, Aerobic OR AB Aerobic Exercises OR AB Aerobic Exercise OR AB Jogging OR AB Walking OR AB Yoga OR AB Ambulation OR AB Swimming OR AB Dancing OR AB Cycling OR AB Resistance OR AB Pilates OR AB Stretching OR AB Tai Chi OR AB Qigong OR AB Mindfulness meditation OR AB gym OR AB fitness OR AB workout OR AB recreation OR AB cardio OR AB relaxation OR AB jog OR AB strength training OR AB Resistance training OR AB Exercise |
| #2 | AB Care, Perinatal OR AB Gestation OR AB Pregnancies OR AB Care, Antenatal OR AB Antenatal Care OR AB Care, Prenatal OR AB Women, Postpartum OR AB Postpartum OR AB Postpartum Women OR AB Puerperium OR AB Care, Postnatal OR AB Postpartum Care OR AB Postpartum Program OR AB Peripartum Periods OR AB Peripartums OR AB Peripartum; Period OR AB Peripartum Women OR AB pre-natal OR AB gestation OR AB parturition OR AB childbirth OR AB antepartum OR AB peripartum OR AB childbearing OR AB prenatal OR AB pregnant OR AB post-partum OR AB post-natal OR AB postnatal OR AB antenatal OR AB Perinatal Care OR AB Pregnancy OR AB Postpartum Period OR AB Prenatal Care OR AB Postnatal Care OR AB Peripartum Period |
| #3 | AB Sleep OR AB Sleep Initiation and Maintenance Disorders OR AB Sleep Quality OR AB Dyssomnias OR AB Sleeping OR AB Sleep Habit OR AB Habits, Sleep OR AB SleeplessnessOR AB Insomnia DisordersOR AB Insomnia DisorderOR AB InsomniasOR AB Secondary InsomniaOR AB Insomnia, SecondaryOR AB Transient InsomniaOR AB Insomnia, ReboundOR AB Psychophysiological InsomniaOR AB Disorders of Initiating and Maintaining SleepOR AB DIMSOR AB Early AwakeningOR AB Insomnia, PrimaryOR AB Primary InsomniaOR AB Chronic InsomniaOR AB Sleep Initiation DysfunctionOR AB Dysfunction, Sleep InitiationOR AB Sleep Initiation DysfunctionsOR AB Nonorganic InsomniaOR AB Quality, SleepOR AB Sleep QualitiesOR AB Qualities, SleepOR AB Adjustment Sleep DisorderOR AB Sleep DisorderOR AB Sleep Disorder, Limit-SettingOR AB Sleep Disorders, ExtrinsicOR AB Sleep Disorders, ExtrinsicOR AB Environmental Sleep DisorderOR AB poor sleepOR AB sleep problemOR AB sleep disturbanceOR AB sleep initiationOR AB sleep maintenanceOR AB sleep restrictionOR AB sleep hygieneOR AB sleep latencyOR AB sleep durationOR AB sleep efficiencyOR AB daytime dysfunctionOR AB sleep outcomeOR AB sleep characteristicsOR AB difficulty falling asleepOR AB restless sleepOR AB awakeningsOR AB snoringOR AB diurnal sleepOR AB diurnal tirednessOR AB sleepinessOR AB sleep dysfunctionOR AB sleep healthOR AB sleep timeOR AB sleep patternOR AB sleep parameters |
| #4 | AB randomized controlled trial OR AB placebo OR AB randomized |
| #5 | A#1 AND #2 AND #3 AND#4 |

**Table 6 Search strategies of EBSCO**

| **steps** | **Search strategies [Search deadline (August 5, 2025)]** |
| --- | --- |
| #1 | abstract(exercise OR physical activity OR fitness OR aerobic training OR strength training OR resistance training OR Sport OR Aerobics OR Training OR Trainings OR Jogging OR Walking OR Ambulation OR Yoga OR Swimming OR Dancing OR Cycling OR Resistance OR Pilates OR Stretching OR Tai Chi OR Qigong OR gym OR workout OR recreation OR cardio OR relaxation OR run OR jog) |
| #2 | abstract(Perinatal Care OR Pregnancy OR Prenatal Care OR Postpartum Period OR Postnatal Care OR Peripartum Period OR perinatal OR antenatal OR postnatal OR post-natal OR postpartum OR post-partum OR pregnan OR pregnant OR prenatal OR childbearing OR peripartum OR puerperium OR antepartum OR parturition OR childbirth OR gestation OR pre-natal) |
| #3 | [abstract(Sleep OR Sleep Quality OR Dyssomnias OR sleeping OR dysomnia OR daytime dysfunction OR difficulty falling asleep OR awakenings OR snoring OR diurnal tiredness OR sleepiness OR insomnia OR sleeplessness)](https://www.proquest.com/recentsearches.recentsearchtabview.recentsearchesgridview.scrolledrecentsearchlist.checkdbssearchlink:rerunsearch/3411467AA8684958PQ/None/$N?_csrf=d7623f61-5ac3-4980-acba-fb7df6b182cf&t:ac=RecentSearches) |
| #4 | abstract(randomized or random or placebo) |
| #5 | A#1 AND #2 AND #3 AND#4 |

**Table 7 Search strategies of CNKI and VIP Information**

| **steps** | **Search strategies [Search deadline (August 5, 2025)]** |
| --- | --- |
| #1 | Exercise + Practice + Physical activity + Aerobic exercise + Training + Yoga + Tai Chi + Pilates + Walking + Cycling + Swimming + Running + Qigong |
| #2 | Perinatal period + Pregnancy + Antenatal + Postnatal + Gestation + Pregnant women |
| #3 | Sleep + Sleep disorder + Sleep quality + Insomnia + Obstructive sleep apnea + Restless legs syndrome + Sleep breathing disorder |
| #4 | Randomized controlled trial + Randomized + RCT |
| #5 | A#1 AND #2 AND #3 AND#4 |
