# Supplementary material for: Optimal types and doses of exercise for improving sleep quality in perinatal women: a systematic review and network meta-analysis based on randomized controlled trials
Source: BMC Pregnancy Childbirth. 2026 Jan 22;26:168. doi: 10.1186/s12884-026-08673-6 (PMC12911199; doi:10.1186/s12884-026-08673-6)
Supplement: Supplementary file 3 — Supplementary Material 3. [file 12884_2026_8673_MOESM3_ESM.docx]

**Figure**


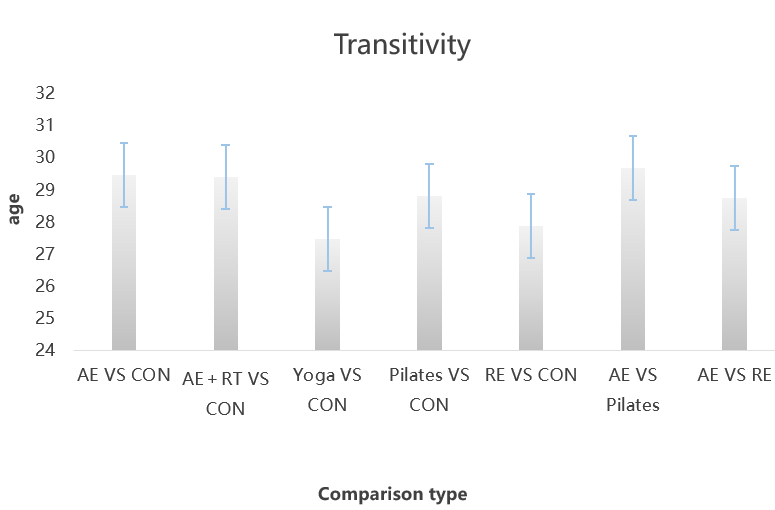


**The consistency of the age distribution of participants in different exercise interventions**
